# Promoting Molecular Exchange on Rare-Earth Oxycarbonate Surfaces to Catalyze the Water–Gas Shift Reaction

**DOI:** 10.1021/jacs.2c10326

**Published:** 2023-01-19

**Authors:** Lu-Lu Zhou, Shan-Qing Li, Chao Ma, Xin-Pu Fu, Yi-Shuang Xu, Wei-Wei Wang, Hao Dong, Chun-Jiang Jia, Feng Ryan Wang, Chun-Hua Yan

**Affiliations:** † Key Laboratory for Colloid and Interface Chemistry, Key Laboratory of Special Aggregated Materials, School of Chemistry and Chemical Engineering, Shandong University, Jinan250100, China; ‡ School of Materials and Environmental Engineering, Chizhou University, Chizhou247000, China; § College of Materials Science and Engineering, Hunan University, Changsha410082, China; ∥ Beijing National Laboratory for Molecular Sciences, State Key Lab of Rare Earth Materials Chemistry and Applications, PKU-HKU Joint Lab in Rare Earth Materials and Bioinorganic Chemistry, Peking University, Beijing100871, China; ⊥ Department of Chemical Engineering, University College London, LondonWC1E 7JE, U.K.

## Abstract

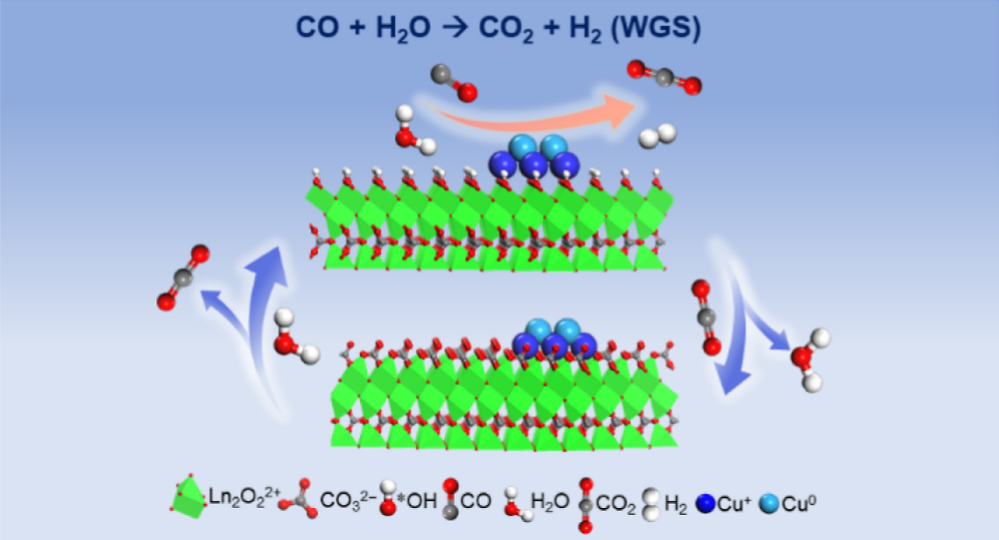

It is highly desirable to fabricate an accessible catalyst surface that can efficiently activate reactants and desorb products to promote the local surface reaction equilibrium in heterogeneous catalysis. Herein, rare-earth oxycarbonates (Ln_2_O_2_CO_3_, where Ln = La and Sm), which have molecular-exchangeable (H_2_O and CO_2_) surface structures according to the ordered layered arrangement of Ln_2_O_2_^2+^ and CO_3_^2–^ ions, are unearthed. On this basis, a series of Ln_2_O_2_CO_3_-supported Cu catalysts are prepared through the deposition precipitation method, which provides excellent catalytic activity and stability for the water–gas shift (WGS) reaction. Density functional theory calculations combined with systematic experimental characterizations verify that H_2_O spontaneously dissociates on the surface of Ln_2_O_2_CO_3_ to form hydroxyl by eliminating the carbonate through the release of CO_2_. This interchange efficiently promotes the WGS reaction equilibrium shift on the local surface and prevents the carbonate accumulation from hindering the active sites. The discovery of the unique layered structure provides a so-called “self-cleaning” active surface for the WGS reaction and opens new perspectives about the application of rare-earth oxycarbonate nanomaterials in C1 chemistry.

## 1 Introduction

In heterogeneous catalysis, it is of significant essentiality to accelerate the reaction process through designing the active catalyst surface to promote the activation of reactant molecules and favor the desorption of product molecules.^1−3^ There is a dynamic equilibrium during the catalytic reaction between the reactants and products on the surface of catalysts; therefore, promoting the equilibrium shift based on Le Chatelier’s principle on the local surface is highly imperative.^4,5^ For decades, a widely concerned issue is whether the catalyst configuration is conducive to the adsorption and activation of reactants,^6−8^ while the desorption of products and the exchange between the reactant dissociates and product adsorbates to boost the equilibrium movement is relatively less investigated.^9^ Accordingly, fabricating an accessible and efficient catalyst surface that combines the advantages of efficacious dissociation of the reactant and desorption of the product is of great significance.

The WGS reaction is a crucial industrial process for hydrogen production, which is a key route to provide sustainable energy. Thus, it is vital to facilitate the forward movement of the reaction.^10−13^ It is widely known that there is competitive adsorption of CO_2_ and H_2_O molecules on the surface of the catalysts for the WGS reaction, and H_2_O dissociation is generally regarded as the rate-determining step.^14−16^ Thus, achieving an effective circulation of CO_2_ and H_2_O in the micro-reaction interface space and optimizing the local equilibrium concentration of each molecule involved in the reaction play the key role in increasing the reaction rate. In the past decades, oxide materials, including CeO_2_,^12,16,17^ FeO_*x*_,^10,11,18^ TiO_2_,^9,15,19^ and so forth, have been commonly employed as the matrix for the supported catalysts. However, owing to the formation of carbonate species, CO_2_ is not discharged effectively. In addition, the accumulation of surface carbonates blocks the active sites, which has always been a tough issue.^20−23^ Accordingly, designing and fabricating an ideal catalyst that separates the H_2_O/CO adsorption site and the CO_2_ desorption site is the optimal solution.

**Figure 1:**
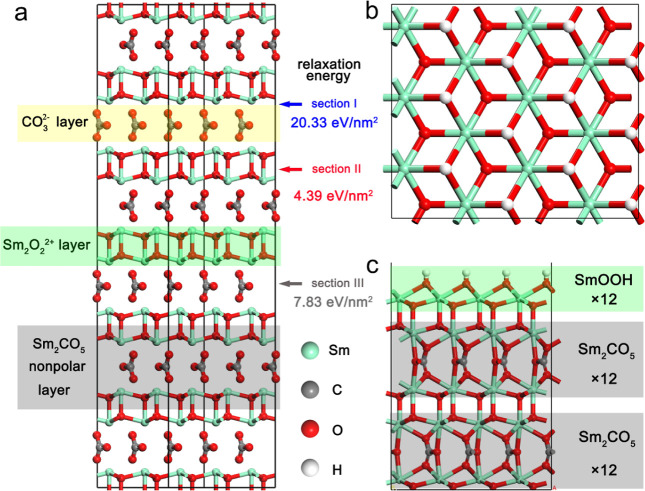
Structure of Sm_2_O_2_CO_3_ calculated by DFT. (a) Layer structure of the Sm_2_O_2_CO_3_ supercell. The Sm–O bonds are hidden in order to see the layering principle better. The Sm_2_O_2_^2+^ and CO_3_^2–^ layers are parallel to the {001} plane. The calculations suggest that section II is the advantaged position when exposed crystal surfaces are generated from the crystal Sm_2_O_2_CO_3_. (b) Top view of the hydroxylated {001} Sm_2_O_2_CO_3_ surface (the inner atoms are concealed). (c) Main view of the hydroxylated {001} Sm_2_O_2_CO_3_ surface, which contains two complete Sm_2_O_2_CO_3_ layers and one hydroxylated Sm_2_O_2_^2+^ layer.

Our strategy lies in a facile and available conversion between the hydroxyl groups dissociating from the reactant H_2_O and the inherent carbonate layer on the catalyst surface, preventing CO_2_-forming carbonate species from blocking the active site. Those structures can be found in light rare-earth oxycarbonates (Ln_2_O_2_CO_3_, Ln = La, Sm, etc) with a hexagonal crystal phase, which demonstrate the layered structure that the carbonate groups (CO_3_^2–^) arranged between Ln–O bilayers (Ln_2_O_2_^2+^). As shown in Figure 1a, such a structure provides a convenient surface for the exchange of molecules (i.e., CO_2_ and H_2_O).^24^ In addition, as a favorable metal for the adsorption of CO molecules, Cu is often used as the active metal to catalyze the WGS reaction. Hence, Ln_2_O_2_CO_3_ may have the unheeded potential for catalyzing the WGS reaction, in which the Ln_2_O_2_^2+^ layer adsorbs H_2_O and then displaces the CO_3_^2–^ layer to release CO_2_. In this work, the Ln_2_O_2_CO_3_ (Ln = La and Sm) is prepared by a controlled hydrothermal approach. Cu species are subsequently loaded on the surface of Ln_2_O_2_CO_3_ through the deposition–precipitation process. The as-prepared 5Cu/Sm_2_O_2_CO_3_ catalyst exhibits excellent activity, following a reaction rate of 1711 μmol_CO_ g_Cu_^–1^ s^–1^ at 300 °C, which is at least nearly 1 order of magnitude higher than other previously reported copper-based catalysts. The combination of density functional theory (DFT) calculations and systematic experimental characterizations confirm the interchangeability of H_2_O and CO_2_ through the exchange of hydroxyl and carbonate on the surface of Ln_2_O_2_CO_3_, which is in favor of H_2_O dissociation and CO_2_ desorption while giving impetus to the equilibrium shifting of the WGS reaction. Meanwhile, the uniformly dispersed Cu^+^ species on the Ln_2_O_2_CO_3_ surface promote the impactful adsorption of CO molecules. The use of Ln_2_O_2_CO_3_ with the layered structure as a catalyst support provides a new strategy for the fabrication of highly active catalysts for the WGS reaction and opens new opportunities in C1 chemistry, such as CH_4_ combustion, CO_2_ reduction, and methanol synthesis.

## 2 Experimental Methods

### 2.1 Catalyst Preparation

#### 2.1.1 Preparation of the Samarium Oxycarbonate Support

The Sm_2_O_2_CO_3_ nanorods (NRs) were synthesized by a modified hydrothermal method.^25^ Typically, 18 mmol of Sm(NO_3_)_3_·6H_2_O were dissolved in 360 mL of deionized water. Then, the pH of the solution was adjusted to 12 by the addition of 10 wt % NaOH solution. Next, the mixed solution was moved into several 100 mL stainless-steel Teflon-lined autoclaves and reacted on a temperature program of 120 °C for 12 h. The obtained mixture was washed three times with deionized water and once with ethanol, and then dried at 80 °C for 3 h. The obtained product was calcined at 450 °C for 4 h in the tube furnace.

#### 2.1.2 Preparation of the Lanthanum Oxycarbonate Support

The La_2_O_2_CO_3_ nanorods (NRs) were synthesized by the hydrothermal method.^26^ First, 14.4 g of NaOH dissolved in 40 mL deionized water was separately loaded into six 100 mL Teflon bottles prepared in advance and stirred for 15 min. Then, 1.3 g of La(NO_3_)_3_·6H_2_O was dissolved in 20 mL of deionized water. The solution was added to the above NaOH solution and continuously stirred for another 15 min. Then, the mixed solution was moved to six stainless-steel autoclaves and reacted on a temperature program of 100 °C for 24 h. The products were divided by centrifugation, washed with deionized water and ethanol, and then, the precipitates were dried overnight at 70 °C in an oven and calcined at 450 °C for 4 h in a tube furnace.

#### 2.1.3 Preparation of the Reference Alumina Support

The support of Al_2_O_3_ nanobelts (NBs) was also prepared by a hydrothermal method.^27^ First of all, 3.22 g Al(NO_3_)_3_·9H_2_O and 4.6 g CO(NH_2_)_2_ were added to the 60 mL of distilled water. Next, the above-mixed solution was loaded into a 100 mL stainless Teflon-lined autoclave and kept at 100 °C for 48 h. Next, the products were washed and centrifuged with distilled water and ethanol and dried at 80 °C for 10 h. The final samples were obtained by calcining at 600 °C for 2 h.

#### 2.1.4 Preparation of the Reference Ceria Support

Reference support CeO_2_ nanorods were synthesized in the same way as Sm_2_O_2_CO_3_ nanorods, except that the precursor was replaced with Ce(NO_3_)_3_·6H_2_O (7.8 g).

#### 2.1.5 Preparation of *x* wt % Cu/Sm_2_O_2_CO_3_ Catalysts

The *x* wt % Cu/Sm_2_O_2_CO_3_ samples were prepared via the deposition–precipitation (DP) method.^28^ To begin with, 0.5 g of the above support, Sm_2_O_2_CO_3_ NRs were dissolved in 25 mL of ultrapure water under continuous stirring. Then, 0.5 mol/L of Na_2_CO_3_ solution and a corresponding volume of 0.1 mol/L of Cu(NO_3_)_2_·3H_2_O solution were added into the Sm_2_O_2_CO_3_ solution simultaneously until Cu(NO_3_)_2_·3H_2_O solution was completely dripped, and the final solution pH was 9. Next, the mixture was aged for 1 h after stirring for 30 min at RT. Then, the obtained precipitation was washed and filtered with 1 L of ultrapure water. Finally, the *x* wt % Cu/Sm_2_O_2_CO_3_ catalysts were obtained after drying at 70 °C for 10 h.

#### 2.1.6 Preparation of *x* wt % Cu/Al_2_O_3_ and *x* wt % Cu/CeO_2_ Catalysts

The synthesis method of reference Cu/Al_2_O_3_ and Cu/CeO_2_ catalysts was the same as Cu/Sm_2_O_2_CO_3_ with the DP method.

### 2.2 Characterization of Catalysts

#### 2.2.1 Inductively Coupled Plasma Atomic Emission Spectroscopy

Inductively coupled plasma atomic emission spectroscopy (ICP–AES) of copper content was performed on an IRIS Intrepid II XSP instrument.

#### 2.2.2 Transmission Electron Microscopy

The transmission electron microscopy (TEM) and high-resolution TEM (HRTEM) images were obtained on an FEI Tecnai G^2^ F20 microscope instrument working at 200 kV. The high-angle annular dark-field scanning transmission electron microscopy (HAADF-STEM) images were conducted using a JEOL ARM200F microscope, which is equipped with a probe-forming spherical-aberration corrector.

#### 2.2.3 N_2_-Sorption Measurement

The data was tested on a Builder SSA-4200 physisorption instrument at 77 K. The samples were pretreated at 473 K for 6 h under vacuum before the test. The specific surface area of samples was calculated by the Brunauer–Emmett–Teller method.

#### 2.2.4 X-ray Powder Diffraction Investigations

The data was acquired on the PANalytical X’Pert3 type X-ray powder diffractometer (λ_Cu.Kα_ = 0.15418 nm), which worked at 40 kV and 40 mA, and the collection time of ex situ XRD was 1 h for *x* wt % Cu/Sm_2_O_2_CO_3_ samples and 8.5 min for reference catalysts.

#### 2.2.5 N_2_O Chemisorption Investigations

The data were obtained on a Builder PCSA-1000 instrument to determine the Cu dispersion. First, the samples were treated at 300 °C for 30 min and heated and reduced in the H_2_ atmosphere until 400 °C. After that, the gas was switched to N_2_O for 1 h at room temperature to oxidize the Cu species on the surface, and the same reduction operation was performed subsequently. The hydrogen consumptions of the two times were labeled as *A*_1_ and *A*_2_ and then the dispersion (*D*) of Cu species was calculated based on the formula of *D* = 2*A*_2_/*A*_1_ × 100%.

#### 2.2.6 Attenuated Total Reflectance Fourier Transform Infrared Spectroscopy Investigations

The data were acquired on a Thermo-Nicolet iS50 Fourier transform infrared (FTIR) spectrometer. The acquisition spectra were collected in the range from 400 to 4000 cm^–1^ with the KBr beam splitter.

#### 2.2.7 Temperature Programmed Reduction by Hydrogen

The data were obtained on the same Builder PCSA-1000 instrument. Before the experiments, the catalysts (50 mg) were preprocessed under air at 300 °C for 30 min and purged in pure Ar at RT. Then, the catalysts were heated to 800 °C under the 5% H_2_/Ar atmosphere with a heating rate of 10 °C·min^–1^.

#### 2.2.8 Ex Situ and Quasi In Situ X-ray Photoelectron Spectrometry Investigations

The data were acquired on a Thermo Scientific ESCALAB Xi^+^ equipped with a high-temperature reaction tank (FERMI). For the in situ test, the samples could directly enter the X-ray photoelectron spectroscopy (XPS) host through the transmission system for measurement after pretreatment.

#### 2.2.9 In Situ FTIR Spectroscopy Investigations

The data were obtained on an ultrahigh vacuum (UHV) apparatus with an FTIR spectrometer and a multichamber UHV system. The prepared samples were preprocessed in an H_2_ atmosphere at 300 °C for 30 min and then CO was let in with desired pressure at –143 °C.

#### 2.2.10 In Situ Diffuse Reflectance Infrared Fourier Transform Spectroscopy Investigations

The data were acquired on a Bruker Vertex 70 FTIR spectrometer with a mercury–cadmium–telluride (MCT) detector, which worked in a liquid nitrogen atmosphere. Prior to collecting the spectra, the catalyst (30 mg) was treated with an Ar atmosphere at 300 °C for 30 min; after that, the switching experiment of 2% CO_2_/Ar–3% H_2_O/Ar–2% CO_2_/Ar was carried out, and the spectra between 1000 and 4000 cm^–1^ were collected.

#### 2.2.11 Mutual Substitution of Hydroxyl and Carbonate on Catalyst Surface Investigations

The data was obtained on an online mass spectrometer (LC-D200M, TILON, MS). After the catalysts were treated at 300 °C during Ar gas for 30 min, there were three test modes. The first experiment was to collect CO^16^O^16^, CO^16^O^18^, and CO^18^O^18^ signals during the cycle switching between O^18^-labeled H_2_O^18^ and CO_2_; the second experiment was to collect C^12^O_2_ and C^13^O_2_ signals during the cycle switching between C^13^-labeled C^13^O_2_ and H_2_O, and the third experiment was to introduce a H_2_O/Ar or pure Ar atmosphere on the surface of the catalysts for 3 h and then switch to pure Ar for the temperature-programed experiment (RT–800 °C). CO_2_ signals were collected during the whole process.

#### 2.2.12 Temperature-Programmed Surface Reaction Investigations

The data was acquired on an online mass spectrometer. The catalyst (100 mg) was activated at 300 °C for 30 min under a 5% H_2_/Ar atmosphere, then lowered to RT in the same atmosphere, and then purged in the pure Ar atmosphere. Then, the 2% CO/3% H_2_O/Ar atmosphere was introduced at 300 °C for 1 h, and the signals of CO, H_2_O, CO_2_, and H_2_ were recorded to confirm the ratio of CO_2_ and H_2_ and create a real in situ WGS reaction atmosphere. Next, the sample was exposed to pure Ar at 300 °C for 120 min to remove the excess gaseous H_2_O. Then, the H_2_O/Ar atmosphere was introduced at 250 °C for around 100 min. During this period, the signal of H_2_ was also recorded to determine whether H_2_O was dissociated. After that, the high-temperature purging was continued to get rid of the gaseous H_2_O under the Ar atmosphere, only leaving the surface hydroxyl groups. Then, the temperature was lowered to RT with the same atmosphere, and 2% CO/Ar was introduced from RT to 300 °C with a heating rate of 10 °C·min^–1^ and kept at 300 °C for 60 min; the signal from CO_2_ and H_2_ was collected during this period. All gas flows are 30 mL·min^–1^.

#### 2.2.13 DFT Calculations

The periodic structures were modeled using the Vienna ab initio simulation package.^29,30^ The Perdew–Burke–Ernzerhof functional and the projector augmented-wave method were applied to the spin-unrestricted optimizations.^31,32^ The plane wave cut-off energy was set to 400 eV, and the convergence criteria of structural optimization were that all the forces are smaller than 0.03 eV/Å. The composite catalyst model comprised a two-layer Sm_2_CO_5_ {001} slab (the bottom layer was fixed), one SmOOH layer, and four Cu atoms. The lattice parameters of the surface were *a* = 13.5252 Å, *b* = 11.7132 Å, *c* = 34.3707 Å, and α = β = γ = 90°, and the chemical formula of the catalyst model was Sm_60_C_24_O_144_H_9_Cu_4_. The binding force between the Cu_4_ cluster and the surface was Sm–O–Cu chemical bonds.

### 2.3 Catalytic Measurements and Kinetic Tests

The activity of the catalysts was tested with a WGS reaction in a fixed-bed flow reactor. 100 mg of the sample was packed into a quartz tube and reduced with a 5% H_2_/Ar flow at 300 °C for 30 min. For the stability test, the sample only needed 50 mg. After Ar purging, the mixture of 2% CO/10% H_2_O/N_2_ was introduced, where the H_2_O was pushed in by a pump. The total gas hourly space velocity (GHSV) was 42,000 mL g^–1^ h^–1^ for reactivity and 84,000 mL g^–1^ h^–1^ for stability. We collected the concentration of outlet gases, CO and CO_2_, from 150 to 350 °C through a Gasboard 3500 IR spectrometer. We used the CO conversion during the reaction as a standard for WGS reaction activity. The specific calculation formula is as follows
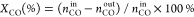
1

### 2.4 Reaction Order and Apparent Activation Energy

The reaction order and apparent activation energy *E*_a_ were tested with the same reactor in catalytic measurements. A suitable amount of sample was weighed to hold CO conversion rates of 5–15% at the target temperatures.

## 3 Results and Discussion

### 3.1 Structure of Sm_2_O_2_CO_3_ and the Ability of Its Surface to Exchange Hydroxyl and Carbonate

We first study the structure of Sm_2_O_2_CO_3_ through DFT calculations and simulation models, and computational details are shown in “Computational Methods” of Supporting Information. As illustrated in Figure 1a, the Sm_2_O_2_CO_3_ crystal presents an alternation of a positively charged Sm_2_O_2_^2+^ layer and a negatively charged CO_3_^2–^ layer. In order to discuss the exposed structure of the Sm_2_O_2_CO_3_ surface, three potential exposed surfaces of the stable {001} facet are compared. Section I in Figure 1a is located between the Sm_2_O_2_^2+^ and CO_3_^2–^ layers. The separation of positive and negative charges leads to a higher relaxation energy of 20.33 eV/nm^2^. Section II, which is situated at the center of the Sm_2_O_2_^2+^ layer, is the most stable location for avoiding charge separation. Section III divides the carbonate ions into equal proportions, namely, half of the carbonate ions are attached to the upper surface and the other half is adsorbed on the lower surface. The simulated results show that section II is more stable than section III at 4.39 versus 7.83 eV/nm^2^. From the standpoints of relaxation energy and stoichiometric proportion, the nonpolar Sm_2_O_2_CO_3_ layer, shown in Figure 1b, is marked as the elementary entity of the surface model. Further calculations, shown in Figure 1c, indicated that compositions SmOOH would spontaneously arrange on the raw surface, that is, the raw surface (Sm_2_O_2_CO_3_ × 24) + 6 Sm_2_O_3_ + 6 H_2_O → the hydroxylated surface (Sm_2_O_2_CO_3_ × 24 + SmOOH × 12) Δ*G* = −11.66 eV. According to the configurations and energy values in Figures S1–S2 and Tables S1–S5, the hydroxylated Sm_2_O_2_CO_3_ surface has a lower energy value and is more stable. Therefore, the DFT calculations demonstrate that the surface of Sm_2_O_2_CO_3_ is highly susceptible to H_2_O, which is vital for the dissociation of H_2_O and further WGS reaction.

**Figure 2:**
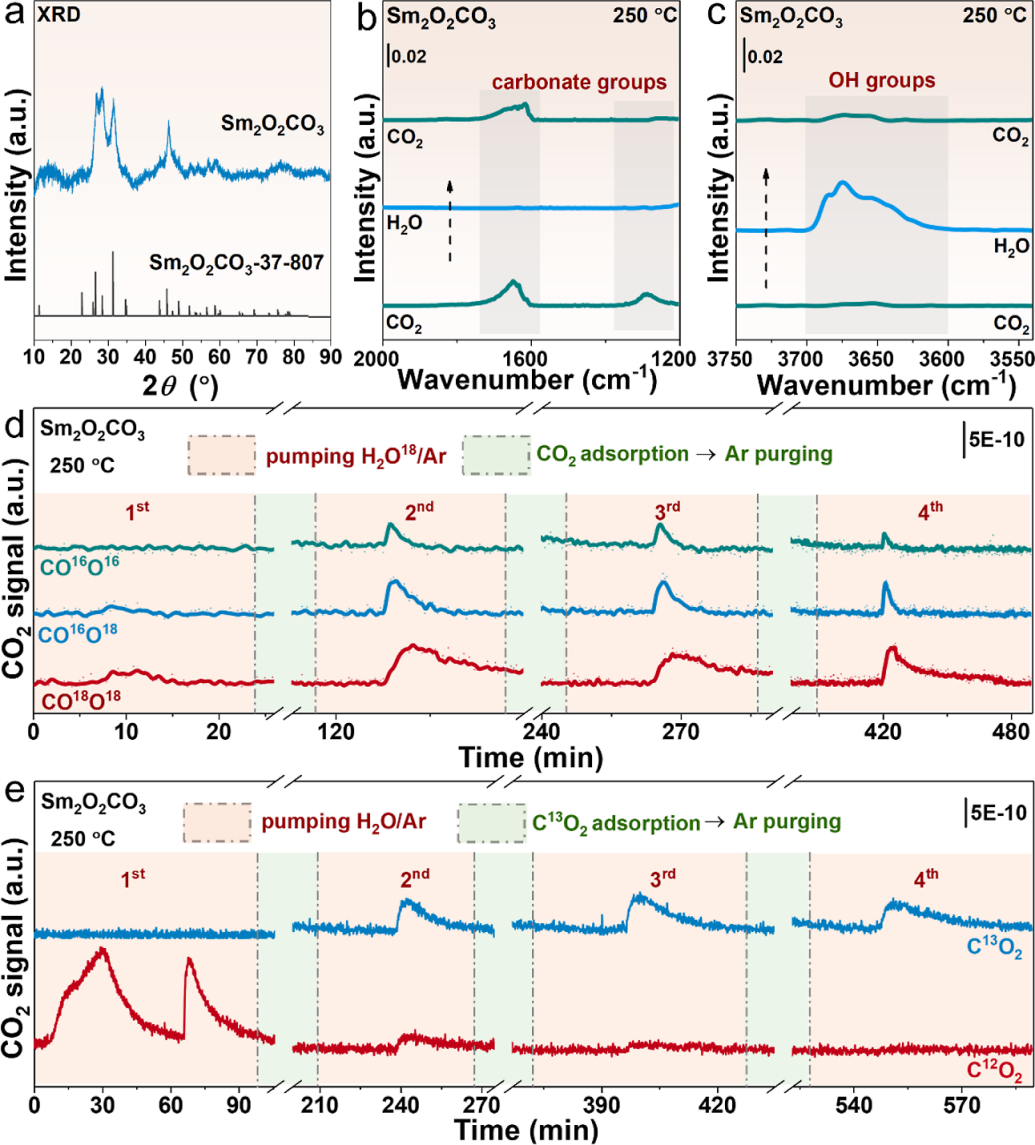
Substitution process of carbonate and hydroxyl species on the surface of Sm_2_O_2_CO_3_. (a) XRD patterns of pristine Sm_2_O_2_CO_3_; Spectral variation of (b) carbonate and (c) OH regions during the switching experiment of CO_2_ and H_2_O; Mass spectrometry signal variation during isotopic gas exchanges of (d) H_2_O^18^ and CO_2_ and (e) C^13^O_2_ and H_2_O.

Note: The carbonate and hydroxyl groups in the formula are adsorbed on the surface of the catalyst.

Sm_2_O_2_CO_3_ is prepared via the hydrothermal method. According to the XRD patterns (Figure 2a), hexagonal Sm_2_O_2_CO_3_ is the dominant phase. Combined with multiple carbonate peaks in attenuated total reflectance FTIR (Figure S3),^33^ the carbonate groups in Sm_2_O_2_CO_3_ are verified. To continue exploring the ability of the Sm_2_O_2_CO_3_ to dissociate H_2_O, the experimental characterizations of in situ DRIFTS and mass spectrometry (MS) are employed. Figure 2b,c shows the spectral variation of carbonate and hydroxyl group regions during the gas exchanges between CO_2_ and H_2_O. In detail, after the introduction of CO_2_ at 250 °C, two distinct peaks attributed to carbonate species appear at 1647 and 1287 cm^–1^,^20,34^ which disappear almost simultaneously when switching to H_2_O, while the hydroxyl groups peak appear within 3650–3700 cm^–1^.^20,24^ Notably, these surface carbonate species (Figure 2b) are different from the intrinsic CO_3_^2–^ in Sm_2_O_2_CO_3_ (1484 and 1370 cm^–1^, Figure S3). More interestingly, when switching back to CO_2_, the peaks of carbonate species reappeared, while the hydroxyl groups disappeared. The above results clearly display the mutual substitution between hydroxyl groups and carbonate on the surface of Sm_2_O_2_CO_3_. Subsequently, as shown in Figure 2d,e, the MS data with isotopic gas exchanges also confirms the exchange capacity. In the exchange experiment of H_2_O^18^ and CO_2_ on the Sm_2_O_2_CO_3_ support surface, various CO_2_ signals emerge after cleaning the surface with Ar gas and H_2_O^18^ exposure (Figure 2d). The phenomenon is believed to be that the O^18^H* generated by the dissociation of H_2_O^18^ replaced the *CO_3_ groups on the surface and successively released CO^16^O^16^, CO^16^O^18^, and CO^18^O^18^, corresponding surface exchange reactions are as follows eqs 2–6
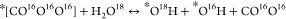
2
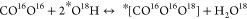
3
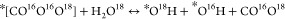
4
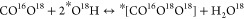
5
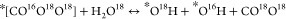
6

In order to further verify the replacement process, CO_2_ adsorption is performed to displace hydroxyl groups and supplement the carbonate layer. After Ar purging, during the second introduction of H_2_O^18^, the corresponding CO_2_ signals reappear as expected. Then, the next two rounds of the CO_2_ adsorption experiment continue to be manipulated. Similarly, the diverse CO_2_ signals are generated again after H_2_O^18^ is introduced, further affirming the continuity of the replacement processes of carbonate and hydroxyl. During the introduction of H_2_O^18^ in each cycle, the generation of the higher intensity of the labeled CO_2_ signal also further confirms that the displaced CO_2_-formed carbonate is efficiently replaced by the *O^18^H generated by the dissociation of H_2_O^18^. In terms of the process of isotopic gas exchanges of C^13^O_2_ and H_2_O (Figure 2e), C^12^O_2_ appears after the first pumping of H_2_O. After that, in the next three rounds of testing, C^13^O_2_ is utilized to replenish the surface carbonate layer, then H_2_O is introduced, and only the signal of C^13^O_2_ appears. C^12^O_2_ only appears in the first cycle, indicating a very efficient exchangeability of *OH to surface *CO_3_. The abovementioned characteristics indicate that the molecular-exchangeable highway of CO_2_ and H_2_O is constructed on the surfaces of rare-earth oxycarbonate.

Thereafter, the MS results of the Ar-temperature-programed experiment with and without H_2_O pretreatment are shown in Figure S4a,b. The obvious CO_2_ signal is generated immediately after the sample is pretreated with H_2_O and the CO_2_ peak at low temperature (designated as surface carbonate) vanishes during the subsequent Ar-temperature-programed process, while the peak of CO_2_ in the high-temperature area (designated as bulk carbonate) remained stable. This is consistent with the exchange of hydroxyl and carbonate layers on the surface of Sm_2_O_2_CO_3_. Furthermore, the 5Cu/Sm_2_O_2_CO_3_ surface exhibits a similar *OH and *CO_3_ exchange capacity to Sm_2_O_2_CO_3_ (Figure S4c). In contrast, there is no CO_2_ produced when H_2_O is introduced for the reference 5Cu/Al_2_O_3_ catalyst (Figure S5), indicating that it does not have the exchange capability. Thus, compared with stable carbonate species on other oxide surfaces,^9,35^ the accessibility of the carbonate layer on the surface of Sm_2_O_2_CO_3_ provides favorable conditions for the exchange with hydroxyl. In conclusion, the combination of theory and experimental study demonstrates that the rare-earth oxycarbonates are naturally beneficial to the dissociation of H_2_O and produce *OH for exchange with the *CO_3_ formed by CO_2_ adsorption, realizing the efficient circulation of reactants and product molecules, which creates favorable conditions for accelerating the progress of the WGS reaction.

### 3.2 Structure and Catalytic Performance of the Copper-Samarium Oxycarbonate Catalysts

**Figure 3:**
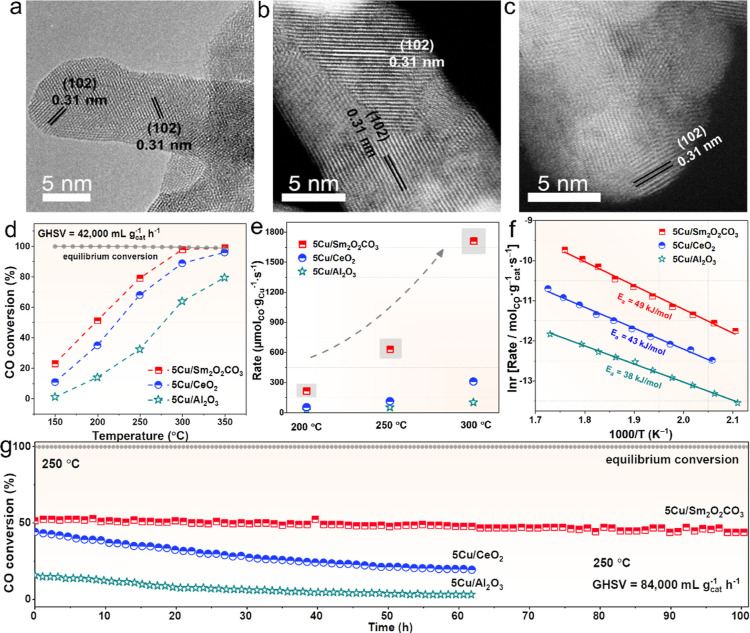
Morphology and catalytic performance over catalysts. (a) HRTEM and (b,c) aberration-corrected HAADF–STEM images of used 5Cu/Sm_2_O_2_CO_3_ catalysts; (d) catalytic performances test (100 mg, GHSV = 42,000 mL g_cat_^–1^ h^–1^), (e) comparison of WGS reaction rates, (f) apparent activation energy value, and (g) stability test for about 100 h of the 5Cu/Sm_2_O_2_CO_3_, 5Cu/CeO_2_, and 5Cu/Al_2_O_3_ catalysts.

After confirming the ability of the support surface to exchange hydroxyl and carbonate, systematic experimental characterizations are performed to identify the morphology and composition of the sample after loading Cu species and the performance for catalyzing the WGS reaction. The actual Cu content based on the ICP result and physical properties of the catalysts are collected in Table S6. The TEM images (Figure S6a,b) unravel that the bare Sm_2_O_2_CO_3_ support presented nanorod-like morphology. Minor changes could be detected after loading 5 wt % Cu on the Sm_2_O_2_CO_3_ surface by the DP method for both fresh (Figure S6c) and used catalysts (Figure S6d). As revealed in the HRTEM and HAADF-STEM images of the used 5Cu/Sm_2_O_2_CO_3_ sample (Figure 3a–c), the characteristic lattice distance with 0.31 nm belongs to the {102} crystal plane of the Sm_2_O_2_CO_3_ matrix, and still, no Cu-related species are observed. This verifies the homogeneous distribution of Cu species on the Sm_2_O_2_CO_3_ surface after the WGS reaction, indicative of the relatively stable metal–support interaction, which can also be confirmed by the EDS elemental mapping images of the used 5Cu/Sm_2_O_2_CO_3_ sample in Figure S7. The XRD data of the 5Cu/Sm_2_O_2_CO_3_ sample, shown in Figure S8, is well in line with the above TEM images, with only the phase of Sm_2_O_2_CO_3_ for the prepared and spent catalysts, and the characteristic diffraction peaks of Cu species are invisible. However, for the spent reference 5Cu/Al_2_O_3_ sample, aggregated Cu particles with a diameter of ∼27 ± 9 nm are visible in the TEM and EDS elemental mapping images, in contrast to the fresh samples with clean surfaces (Figure S9). Similarly, the characteristic peak of metallic Cu is observed in the XRD patterns (Figure S10). Another reference 5Cu/CeO_2_ catalyst is the same as 5Cu/Sm_2_O_2_CO_3_, the Cu species are well dispersed from TEM images (Figure S11), and no peaks for Cu species in the XRD result (Figure S12).

The WGS reaction catalytic performance of the samples is evaluated over the tested temperature range of 150 to 350 °C. The typically irreducible Al_2_O_3_ and reducible CeO_2_ are employed as reference supports for loading Cu species to confirm the superiority of the Sm_2_O_2_CO_3_ support material. As shown in Figure 3d, as expected, the 5Cu/Sm_2_O_2_CO_3_ catalyst exhibits the CO conversion of ∼80% at 250 °C and reaches the equilibrium conversion at 300 °C, with a GHSV of 42,000 mL g^–1^ h^–1^, which outperformed that of Cu/CeO_2_ (∼67%–250 °C) and Cu/Al_2_O_3_ (∼30%–250 °C). Meanwhile, the CO consumption rate of 5Cu/Sm_2_O_2_CO_3_ is 5–6 times that of 5Cu/CeO_2_ and 10–17 times that of 5Cu/Al_2_O_3_ at various temperatures (Figure 3e) and is at least nearly one order of magnitude higher than that of other copper-based samples, which reported in the literature, as shown in Table 1.^17,18,28,36−47^ In addition, these three catalysts demonstrate approximate apparent activation energies (*E*_a_) but diverse prexponential factors values (*A*) in Figure 3f. This reflects the similar reaction mechanism of these catalysts, while the 5Cu/Sm_2_O_2_CO_3_ possesses the most active sites,^48^ which is in accordance with the numerous surface-active hydroxyl sites in Sm_2_O_2_CO_3_, as calculated by DFT. Besides, the kinetic test shown in Figures S13 and S14 also indicates that the pre-exchanged H_2_O molecules directly contributed to the generation of CO_2_ via surface reaction. Furthermore, the 5Cu/Sm_2_O_2_CO_3_ catalyst presents excellent long-term durability at 250 °C under the condition that the CO conversion rate is far from the equilibrium conversion rate and a higher GHSV of 84,000 mL g^–1^ h^–1^, retaining nearly 90% of the original activity within 100 h (Figure 3g). In contrast, the 5Cu/CeO_2_ and 5Cu/Al_2_O_3_ catalysts lose 57% and 81% of their initial activity after the 60 h stability test, respectively. Meanwhile, the apparent catalytic performance test (Figure S15) clearly indicates that the 5Cu/Sm_2_O_2_CO_3_ catalyst possesses the lowest decline rates of the CO conversion rate after the introduction of CO_2_ atmosphere in the catalytic system compared to the other two samples. These results reach an agreement with the abovementioned exchange process, in which the *OH generated by the dissociation of H_2_O on the surface of 5Cu/Sm_2_O_2_CO_3_ sufficiently exchanges the *CO_3_ formed by CO_2_ adsorption, promoting the local equilibrium shifting of the reaction by effectively release of CO_2_. The performance characterization results of the catalysts verify the superiority of the 5Cu/Sm_2_O_2_CO_3_ catalyst, compared to conventional oxide catalysts.

**Table 1:** Comparison of the Reaction Rates Over Various Catalysts

samples	CO/H_2_O	Cu (wt %)^a^	temperature (°C)	rate (μmol_CO_ gCu^–1^ s^–1^)	ref.
5Cu/Sm_2_O_2_CO_3_	1:5	3.8	300	1711	this work
5Cu/GDC (combustion)	1:2	5.0	300	74	[^36^]
Mg_0.52_Cu_0.35_Ce_0.13_(G)	1:3	23.0	300	213	[^37^]
Mg_0.52_Cu_0.35_Ce_0.13_	1:6	23.0	300	361	[^37^]
Cu_0.73_Ce_0.23_	1:6	44.5	300	67	[^37^]
Cu-in-TiO_2_NT	N/A	2.0	300	31	[^38^]
CuZnAl	1:6.7	50.6	300	15	[^39^]
CuO/Fe_2_O_3_	1:1	2.4	330	167	[^18^]
Cu/Al–Fe	1:1	2.3	330	257	[^40^]
5Cu/Sm_2_O_2_CO_3_	1:5	3.8	250	632	this work
Cu/CeO_2_-NP(T)	1:1	5.1	250	219	[^41^]
Cu_10_/SiO_2_	1.1:1	10	250	14	[^42^]
Cu_10_Fe_1·0_/SiO_2_	1.1:1	10	250	19	[^42^]
7.0CuCe-Cube-L	1:5	7	250	18	[^28^]
5Cu/Sm_2_O_2_CO_3_	1:5	3.8	200	216	this work
CuCe–H_2_	1:6.7	14.4	200	21	[^43^]
Cu/CeO_2_-473	1:3	3.4	200	147	[^17^]
CMO-CP	1:2	28.1	200	10	[^44^]
CuO–CeO_2_	1:3.1	6.4	200	2	[^45^]
CuO–Al_2_O_3_	1:3.1	6.4	200	38	[^45^]
CuO–ZnO–Al_2_O_3_	1:3.1	32	200	24	[^45^]
Cu/TiO_2_{001}	1:2	11	200	20	[^46^]
10%Cu–Ce(La)O_*x*_	1:5	10	200	21	[^47^]

a Determined by ICP-AES.

### 3.3 Study on Reactive Cu Species for CO Adsorption

**Figure 4:**
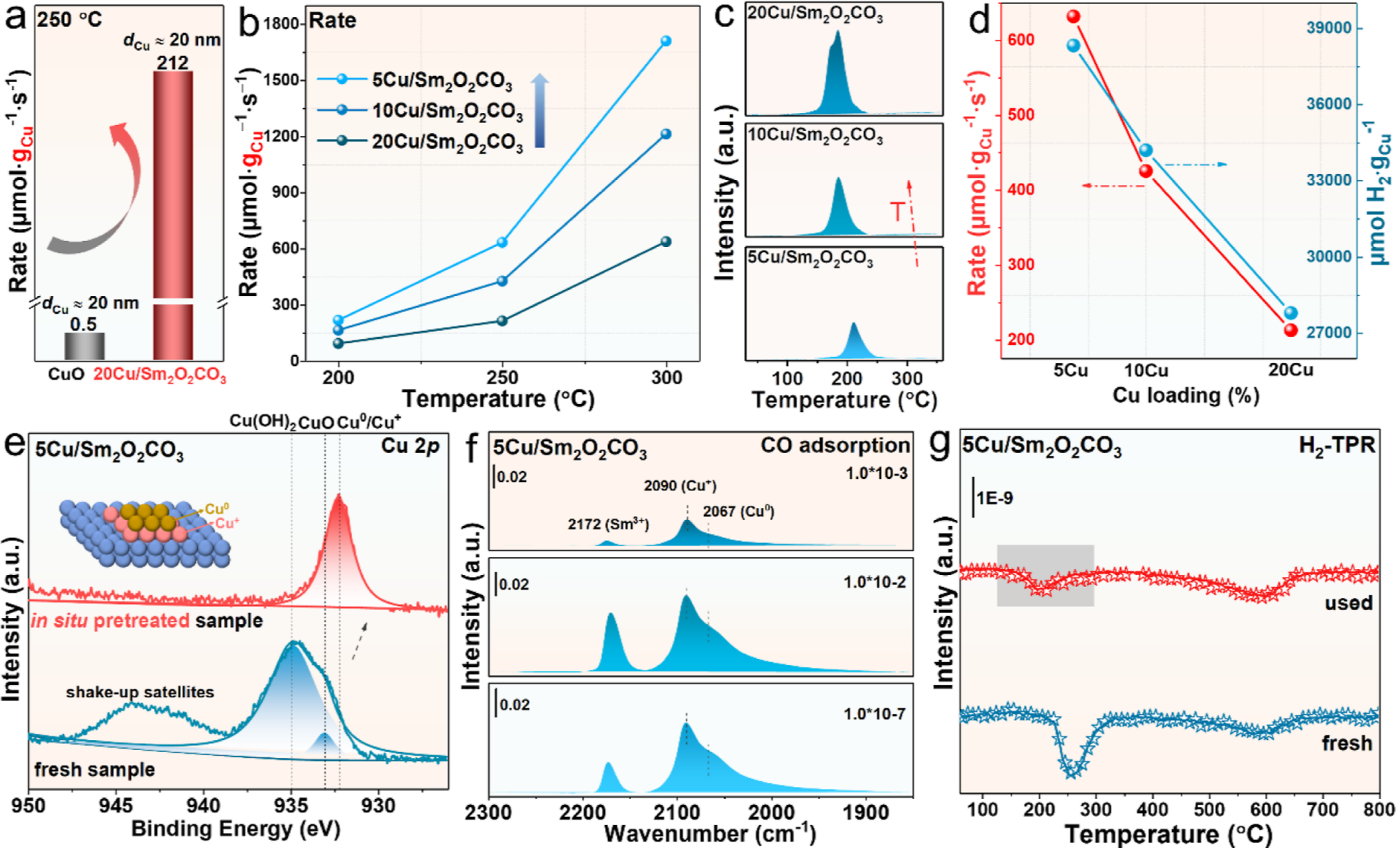
Identification of the active site of the Cu/Sm_2_O_2_CO_3_ catalyst. (a) Reaction rates normalized by Cu weight for CuO and 20Cu/Sm_2_O_2_CO_3_ samples at 250 °C; (b) reaction rates normalized by Cu weight at various temperatures and (c) TCD signals of H_2_-TPR profiles over *x*Cu/Sm_2_O_2_CO_3_ (*x* = 5, 10 and 20) catalysts; (d) reaction rates normalized by Cu weight at 250 °C and calculation of hydrogen consumption normalized by Cu weight from H_2_-TPR for *x*Cu/Sm_2_O_2_CO_3_ samples; (e) ex situ Cu 2*p* XPS spectra of fresh 5Cu/Sm_2_O_2_CO_3_ catalyst and quasi in situ Cu 2*p* XPS spectra of used 5Cu/Sm_2_O_2_CO_3_ catalyst; (f) in situ infrared spectra of 5Cu/Sm_2_O_2_CO_3_ exposed to different CO pressures at −143 °C after H_2_ pretreatment. (g) MS signals of H_2_-TPR profiles of fresh and used 5Cu/Sm_2_O_2_CO_3_ samples.

The status of Cu species is also critical for the adsorption of CO molecules in supported catalysts. To clarify the true active site of adsorption of CO in the WGS reaction, we have prepared a series of *x*Cu/Sm_2_O_2_CO_3_ (*x* = 5, 10, and 20) catalysts with various Cu contents. Meanwhile, bare CuO nanoparticles with diameters of ∼20 nm are controllably synthesized, which are comparable to the size of Cu particles precipitated in the 20Cu/Sm_2_O_2_CO_3_ sample (Figure S16). According to the CO consumption rate normalized by Cu weight (*r*) of these two samples at 250 °C in Figure 4a, it clearly shows that the *r* value of 20Cu/Sm_2_O_2_CO_3_ is much higher than that of bare CuO particles (212 vs 0.5). In addition, from the catalytic activity of *x*Cu/Sm_2_O_2_CO_3_ shown in Figure S17, the reactivity of supported catalysts has been greatly improved compared to bare support and CuO; the results suggest that the WGS reaction might occur preferentially on the interface of samples, and the interaction between the Cu and support enhances the reactivity. Furthermore, the XRD patterns (Figure S18) and the Cu dispersion experiments (Figure S19 and Table S7) reveal that the characteristic peaks of metallic Cu are more obvious, and the Cu dispersions are lower in the samples with a higher Cu content. Moreover, the reaction rate (*r*) normalized by Cu weight in Figure 4b exhibits that the Cu/Sm_2_O_2_CO_3_ samples with lower Cu content have a higher *r* value, which further confirms that the small-sized Cu species with good dispersion at the interface are the better active species.

For the purpose of revealing the redox properties of Cu species in *x*Cu/Sm_2_O_2_CO_3_ samples, H_2_-TPR is performed and shown in Figures 4c and S20. Combining the H_2_-TPR results in the region in 300–800 °C (Figure S20a) and MS signal of CO_2_ in 5Cu/Sm_2_O_2_CO_3_ catalyst during temperature programing under Ar (Figure S20b), the reduction peak below 300 °C is designated as the reduction of Cu species, and the peaks above 300 °C is designated as the decomposition of Sm_2_O_2_CO_3_. Thus, the increase in reduction temperature is accompanied by a decrease in Cu loading, demonstrating the stronger interaction between Cu and support in the samples with a low Cu content (Figure 4c). Correspondingly, the Cu-normalized activity of the samples and the integrated area of peaks in H_2_-TPR are negatively correlated with the Cu content as displayed in Figure 4d. Accordingly, the samples with low Cu content have more Cu species that coordinate with O species, which have preferable WGS reactivity.

Figure 4e displays the ex situ and quasi in situ Cu 2*p* XPS spectra of 5Cu/Sm_2_O_2_CO_3_ with a deconvolution by the Gaussian peak fitting method to confirm the electronic status of Cu species before and after the reaction. According to the collected spectra, the substantial part of the Cu species in the pristine sample exists in the form of Cu(OH)_2_ located at 934.8 eV, with a modicum of CuO located at 933 eV.^49,50^ All these oxidized Cu species are reduced to Cu^+^ or Cu^0^ situated at 932.2 eV in the spent sample,^28^ accompanied by the disappearance of shake-up satellite peaks. For the Sm 3*d* spectra (Figure S21a) obtained from the catalyst surface, the peaks are attributed to the Sm^3+^, which exists stably in the fresh and used samples. As to the C 1*s* spectra (Figure S21b), the two peaks are assigned to the C–C bond and surface carbonates,^51^ which is in line with the carbonate from Sm_2_O_2_CO_3_. The status of Cu species is continuously explored through the in situ infrared spectra at −143 °C with CO adsorption on the *x*Cu/Sm_2_O_2_CO_3_ catalysts after H_2_ pretreatment, which is shown in Figures 4f and S22. Based on the adsorption of CO on diverse Cu species reported in the literature, located at 2120–2140, 2100–2120, and 2000–2100 cm^–1^ are ascribed to Cu^2+^, Cu^+^, and Cu^0^, respectively.^52−54^ After the introduction of different pressures of CO, three CO bands at 2067/2071, 2090/2093, and 2172 cm^–1^ emerge for 5Cu/Sm_2_O_2_CO_3_ (Figure 4f) and 10Cu/Sm_2_O_2_CO_3_ (Figure S22) catalysts, which are assigned to the CO–Cu^0^, CO–Cu^+^, and CO-Sm^3+^, respectively. The intensity of CO adsorption on Cu species increases with the increase of CO pressure, while the adsorption on Sm^3+^ decreases significantly with increasing the degree of vacuum to 1.0 × 10^–7^ mbar, indicating a weak CO-Sm^3+^ bond energy. In situ infrared test reflects the coexistence of Cu^+^ and Cu^0^ in *x*Cu/Sm_2_O_2_CO_3_ samples after reduction pretreatment. Meanwhile, the significant desorption of CO during the process of N_2_ purging (Figure S23) demonstrates that the CO is unable to block the surface sites. Furthermore, the MS signals of H_2_ are recorded during the H_2_-TPR process of the fresh and used 5Cu/Sm_2_O_2_CO_3_ samples, as displayed in Figure 4g, and the results demonstrated that the oxidized Cu species still present in the system after the in situ WGS reaction. Combining the above systematic characterizations, we explore the states of Cu species and the active sites of CO adsorption during the WGS reaction, respectively, and confirm that Cu^+^ and Cu^0^ coexist during the reaction, but Cu^+^ at the interface is the better reactive species.

After determining the adsorption and dissociation sites of CO and H_2_O, we further analyze the dependence of the sample reactivity on the concentration of different reactants and product molecules in the WGS reaction by measuring the reaction order. As shown in Figure S24, we can see that the 5Cu/Sm_2_O_2_CO_3_ catalyst gives a higher absolute value of CO reaction order and a lower absolute value of H_2_O and CO_2_ reaction order compared with the 5Cu/Al_2_O_3_ catalyst. This demonstrates that the surface of the 5Cu/Sm_2_O_2_CO_3_ catalyst has unsaturated CO, which will not cover the active sites, as well as the ability to dissociate H_2_O and desorb CO_2_ efficiently. In addition, the steady-state isotopic transient kinetic analysis (SSITKA) experiments shown in Figures S25–S28 show that the 5Cu/Sm_2_O_2_CO_3_ sample exhibits a lower surface coverage of CO_2_ () and higher surface coverage of CO (*θ*_CO_) compared with the 5Cu/Al_2_O_3_ catalyst, demonstrating that H_2_O molecules are relatively apt to replace generated CO_2_ molecules under reaction conditions and that the desorption of CO_2_ molecules is effective. This further confirms the exchange of H_2_O and CO_2_, as well as its positive role in promoting H_2_O dissociation and CO_2_ desorption and accelerating the WGS reaction.

### 3.4 WGS Reaction Mechanism Study

**Figure 5:**
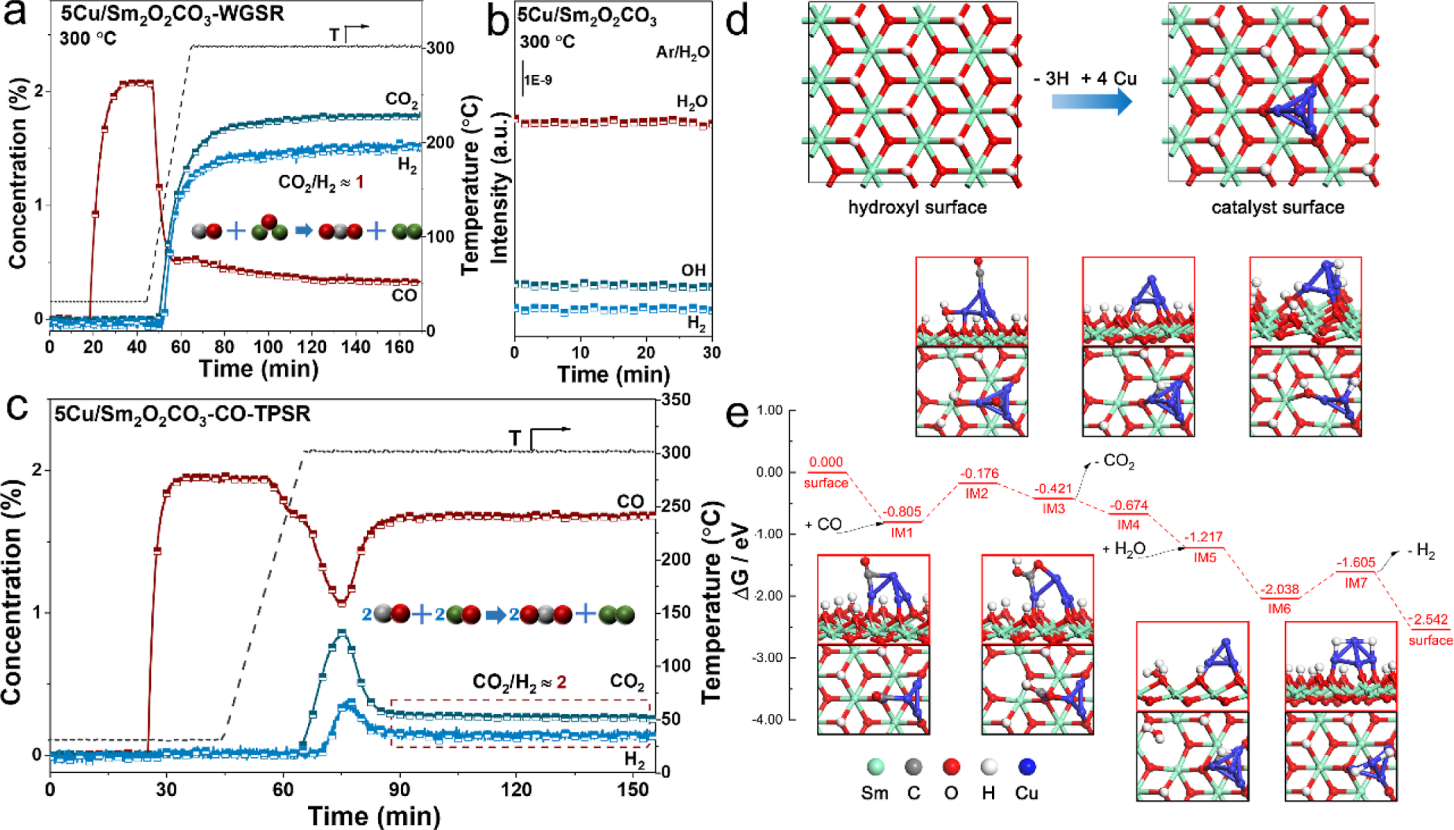
WGS reaction mechanism study of the 5Cu/Sm_2_O_2_CO_3_ catalyst. (a) In situ WGS reaction at 300 °C, (b) experiment of H_2_O dissociation, (c) CO-TPSR of the catalyst, and (d) structure of catalyst surface (top view). (e) Reaction mechanism scheme of the WGS reaction. The structure diagrams with red frames are the main views of intermediate structures, and the diagrams with black frames are top views (the inner atoms are concealed). The Gibbs free energies are calculated at 250 °C, with the partial pressure of CO of 2 kPa and the partial pressure of H_2_O of 10 kPa.

As an important industrial hydrogen production reaction, the mechanism of WGS reaction has always been the focus of attention. At present, two prominent mechanisms of WGS reaction for Cu-based catalysts have been proposed, the redox mechanism and the associative mechanism.^55,56^ It is generally believed that the redox mechanism is that the CO molecules adsorbed on the surface of samples and reacted with the oxygen of the metal-oxide support to form CO_2_, leading to the generation of the oxygen vacancies, which are responsible for the H_2_O dissociation to produce hydrogen. Then, the two hydrogen atoms combined to form hydrogen gas.^14^ In the associative mechanism, the adsorbed CO molecules reacted with the *OH derived from H_2_O dissociation to form the intermediate, which immediately decomposed to generate CO_2_ and H_2_.^57,58^ A TPSR test is conducted to check the mechanism followed by the 5Cu/Sm_2_O_2_CO_3_ sample for catalyzing the WGS reaction. Before verification, we created a realistic catalyst surface through the in situ WGS reaction at 300 °C (Figure 5a). In the calibration concentration curve of the WGS reaction process, the signal ratio of CO_2_ to H_2_ remains stable at 1, which is well in line with the ratio of the CO and H_2_O reaction products (CO + H_2_O = CO_2_ + H_2_). After that, the sample surface is cleaned under Ar gas to remove the gaseous H_2_O molecules, and then H_2_O is reintroduced separately to observe the H_2_ signal. Within 30 min, no H_2_ signal is detected (Figure 5b), making it clear that there is no redox mechanism. Continue to remove gaseous H_2_O molecules below 300 °C in the Ar atmosphere, leaving only surface *OH. After lowering to RT, we switch to 2% CO/Ar for the reaction with *OH and observe the ratio of CO_2_ and H_2_ concentration in the outlet gas. To eliminate the influence of CO_2_ generated by the decomposition of the support, the temperature rise is interrupted and started to maintain after 300 °C because the CO_2_ signal appears in the sample after 300 °C from the experiment (Figure S20b) of temperature programming under Ar. Interestingly, the ratio of generated CO_2_ and H_2_ is perfectly preserved at 2 for 1 h at 300 °C (Figure 5c), proving the associative mechanism of the 5Cu/Sm_2_O_2_CO_3_ catalyst for catalyzing the WGS reaction. The 20Cu/Sm_2_O_2_CO_3_ sample exhibits the same result, which is shown in Figure S29.

In addition, the DFT calculations are also performed to validate the WGS reaction mechanism. To simulate the composite catalyst, four Cu atoms are combined with the hydroxyl surface using the Cu–O bond, as shown in Figure 5d. The WGS reaction mechanism on the catalyst model is composed of eight elementary reactions, as shown in Figure 5e. The WGS reaction started with one CO adsorbed on the Cu cluster (IM1); then, the hydroxide radical on the catalyst surface migrates to the boundary between the Cu cluster and the hydroxyl surface (IM2). The subsequent elementary reaction happens with the formation of the C–O bond, namely, IM3. The first product, CO_2_, is released while the Cu–C and Cu–O bonds are ruptured (IM4). The transient hydroxyl vacancy on the surface is unstable and inclined to capture water molecules (IM5). The adsorbed H_2_O is decomposed into *OH and *H (IM6), and then, the *H combined with the H atom formed in the previous step and emitted H_2_ (IM7). There are six spontaneous elementary reactions, and the other two reactions (the OH-migration and the H-migration, i.e., IM1 → IM2 and IM6 → IM7) have positive Gibbs energies of reaction. Despite the lower thermodynamic equilibrium ratios of IM2 and IM7, the WGS reaction occurs continuously on account of the Gibbs energy of the overall reaction, that is, −2.542 eV. In addition, DFT calculations accompanied by the very high Gibbs energies also exclude the redox mechanism (Table S8), which is in line with the results of experiment. Therefore, the associative mechanism is confirmed for Cu/Sm_2_O_2_CO_3_ to catalyze the WGS reaction with the support of theoretical calculations and experiments. However, it is noteworthy that the calculations have their drawbacks. The molecular formula of the computational model is Sm_60_C_24_O_144_H_9_Cu_4_, and the number of atoms to simulate is much less than that of real catalysts. This means that the simulation will inevitably be affected by the computing capabilities of quantum chemistry software and workstations. There are also limitations of accuracy, such as reducing the overestimated bond energies and increasing the bond lengths, using the method on account of the generalized gradient approximation. The imprecise mesh sampling of phonon frequency calculations can also cause deviations in the chemical potentials of catalysts. In summary, Cu^+^ is in charge of CO adsorption, while Sm_2_O_2_CO_3_ focuses on the dissociation of H_2_O. Here, the adsorbed CO reacts with *OH to form CO_2_ and H_2_O, promoting the WGS reaction process. In addition, the system is successfully extended to Cu/La_2_O_2_CO_3_ catalysts, which exhibit similar properties to Cu/Sm_2_O_2_CO_3_, including the exchange capacity of CO_2_ and H_2_O on the catalyst surface, prominent WGS reactivity, and an associative mechanism for the WGS reaction (Figures S30–S32).

## 4 Conclusions

It is crucial to design and synthesize catalysts whose structures are conducive to the dissociation of the reactant molecules and the desorption of product molecules in the catalytic reactions, further promoting the equilibrium shift. Herein, the Ln_2_O_2_CO_3_ (Ln = La and Sm) structure with an ordered arrangement of metal oxide (Ln_2_O_2_)^2+^ and CO_3_^2–^ layers is prepared by the simple hydrothermal method, and after loading the active metal Cu, it exhibits unexpected activity and excellent durability in the WGS reaction. An interesting phenomenon is observed that the surface carbonate layer and hydroxyl can be interchanged and replenished by CO_2_ and H_2_O, respectively, which facilitates efficient cycling of the reaction and neatly solves the problem of carbonate accumulation and H_2_O dissociation. The proposal of this layered structure containing metal oxide and carbonate provides vast and unforeseen opportunities for the application of rare-earth complex oxides in the field of catalysis.

## Supporting Information Available

Methods of simulation and experimental data, including catalytic reactivity, TEM, XRD, XPS, and in situ DRIFTS results (PDF)
