# Supplementary material for: Promoting Molecular Exchange on Rare-Earth Oxycarbonate Surfaces to Catalyze the Water–Gas Shift Reaction
Source: J Am Chem Soc. 2023 Jan 19;145(4):2252–63. doi: 10.1021/jacs.2c10326 (PMC9896556; doi:10.1021/jacs.2c10326)
Supplement: Supplementary file 1 — ja2c10326_si_001.pdf [file ja2c10326_si_001.pdf]

Supplementary Information for

Promoting Molecular Exchange on Rare-Earth Oxycarbonate Surfaces to Catalyze the Water–Gas Shift Reaction

Lu-Lu Zhou,[1] Shan-Qing Li,[2] Chao Ma,[3] Xin-Pu Fu,[1] Yi-Shuang Xu,[1] Wei-Wei Wang,[1] Hao Dong,[4] Chun-Jiang Jia[1,*] Feng Ryan Wang,[5,*] and Chun-Hua Yan[4,*]

[1]Key Laboratory for Colloid and Interface Chemistry, Key Laboratory of Special Aggregated Materials, School of Chemistry and Chemical Engineering, Shandong University, Jinan 250100, China

[2] School of Materials and Environmental Engineering, Chizhou University, Chizhou 247000, China

[3]College of Materials Science and Engineering, Hunan University, Changsha 410082, China

[4]Beijing National Laboratory for Molecular Sciences, State Key Lab of Rare Earth Materials Chemistry and Applications, PKU-HKU Joint Lab in Rare Earth Materials and Bioinorganic Chemistry, Peking University, Beijing 100871, China

[5]Department of Chemical Engineering, University College London, London WC1E 7JE, UK

*Corresponding author. Email: jiacj@sdu.edu.cn; ryan.wang@ucl.ac.uk; yan@pku.edu.cn

Computational Methods

The Simulation Models and Computational Details. The initiating structure of Sm_2CO_5 was derived from the Crystallography Open Database (COD ID: 1000463. La_2CO_5, P63/mmc, a=b=4.0755; c=15.957; α=β=90°; γ=120°). The reasons of we chose this La_2CO_5 crystal structure were as follow: 1. No corresponding crystal structure found in the database; 2. The X-ray powder diffraction data for Sm_2CO_5 and La_2CO_5 are very similar. After replacing with Sm atoms, the geometric structure of the crystal unit cell was calculated and the final lattice parameters were a=b=3.90440; c=1528901; α=β=90°; γ=120°. The spin-unrestricted PBE simulations were carried out using the Vienna ab Initio simulation package[1,2]. The cutoff energy for the plane wave basis was set to 400 eV. The convergence threshold of the electronic self-consistency was specified as 1.0×10^{-6} eV, and the total energy change of the whole catalyst system between two ionic relaxation steps was designated as less than 0.02 eV. The density-functional-perturbation theory (DFPT) was used to compute second derivatives of the total energy with respect to the position of the ions, and thermal properties were achieved by Phonopy code[3].

Characterization of the catalysts

Steady-state test through the mass spectrometry (MS). The $5Cu/Sm_2O_2CO_3$ catalyst (30 mg, 20−40 mesh) is pretreated with 5% H_2/Ar at 300 °C for 30 min, and then purged with Ar atmosphere. Next, the sample is separately pretreated by four different atmospheres, 2% CO_2/Ar, 10% H_2O/Ar, 10% H_2O^{18}/Ar and 10% D_2O/Ar at 250 °C for 30 min. And then the mixture of reactants with 2% CO/3% H_2O/Ar atmosphere is introduced after Ar purging, while the signals of products are recorded through MS.

Activity test after the introduction of 2% CO_2. The reactants of 2% CO/10% H_2O/N_2 are introduced at 250 °C after the catalysts are pretreated with 5% H_2/Ar at 300 °C for 30 min and purged with Ar atmosphere. The mixture of 2% CO/10% H_2O/N_2 is converted to 2% CO/2% CO_2/10% H_2O/N_2 after 30 minutes.

In-situ DRIFTS study of CO adsorption and desorption. The catalyst (30 mg) is

pretreated with 5% H_2/Ar at 300 °C for 30 min. After the background is obtained in N_2, the gas with the order of 2% CO/He and N_2 is introduced. The spectra acquisition time is 45 s with a resolution of 4 cm^{-1}.

The steady-state isotopic transient kinetic analysis (SSITKA) experiments. The SSITKA technique coupled with mass spectrometry is performed at 250 °C. Prior to the test, the catalysts are activated at 300 °C in 5% H_2/Ar for 30 min, and then the following 4 types sequence of step gas switches: 5% $^{12}CO_2$/He → 5% $^{13}CO_2$/Ar, 5% $^{12}CO_2$/10% H_2O/He → 5% $^{13}CO_2$/10% H_2O/Ar, 0.5% ^{12}CO/He → 0.5% ^{13}CO/Ar and 0.25% ^{12}CO/1% CO_2/50% Ar/He → 0.25% ^{13}CO/1% CO_2/Ar.

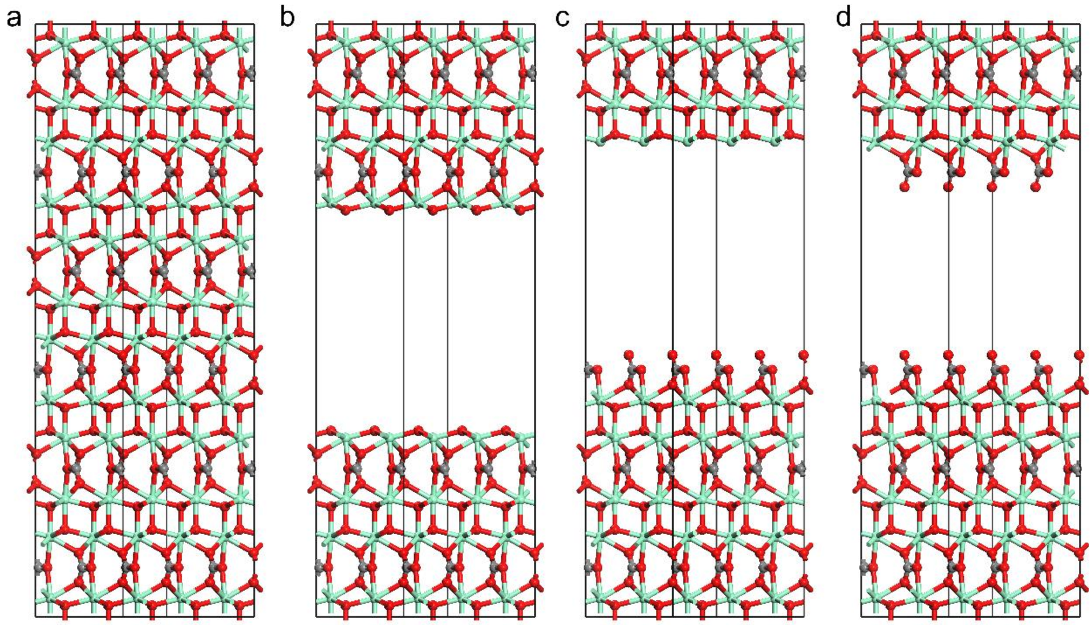

Figure S1. The possible structure of the exposed {001} crystal plane. (a) the supercell structure of Sm_2CO_5; (b) the cross section is located in the interlayer of $Sm_2O_2^{2+}$; (c) the cross section is situated between $Sm_2O_2^{2+}$ and CO_3^{2-} layers; (d) the carbonates are uniformly distributed among upper and lower surfaces. The surface relaxation energies of structure b, c and d are 4.39, 20.33 and 7.83 eV/nm^2, respectively.

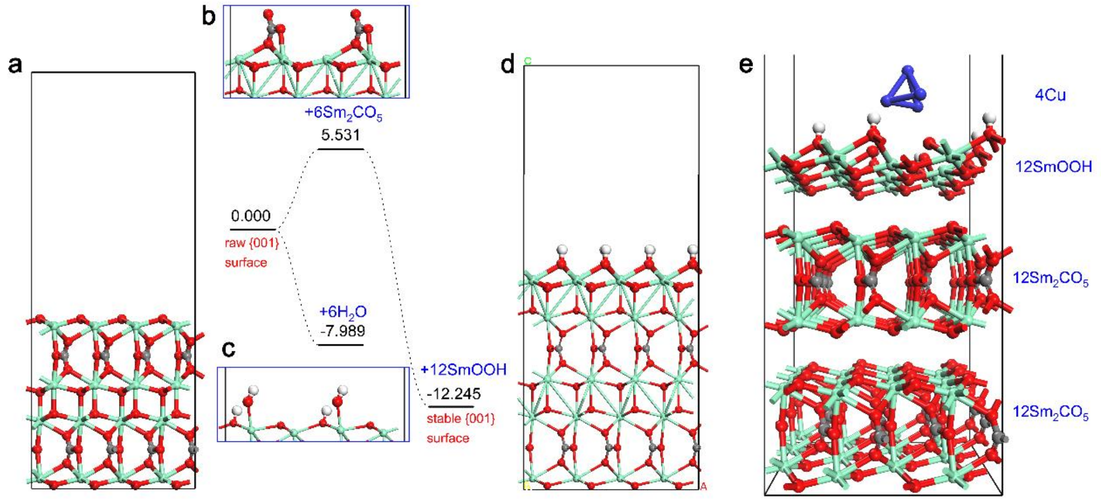

Figure S2. The modeling processes. (a) The raw exposed {001} crystal plane consisted of two $Sm_2O_2CO_3$ layers (12 $Sm_2O_2CO_3$ molecules each), and the selecting rule is listed in the Figure S1; (b) six additional $Sm_2O_2CO_3$ molecules were placed on the raw surface, and the energy (single point energy) change is 5.531 eV; (c) the energy release is 7.989 eV during the six water molecules are loaded on the raw surface. (d) the hydrolytic process of the structure Figure S2b has a significant exothermic effect, and this made structure d quite stable. In this paper, we chose structure d as the model of $Sm_2O_2CO_3$ {001} crystal plane. The composition of the model is 24 $Sm_2O_2CO_3$ and 12 SmOOH; (e) for modeling the Cu/$Sm_2O_2CO_3$ catalyst the model e is raised, in which 4 copper atoms (tetrahedron configuration) are bonded with the surface oxygen atoms on the model d. Three hydrogen atoms are deleted in order to simulate the Cu—O chemical bond, and the atomic coordinates of this catalyst model, i.e. model e, are listed in the Table S1.

The bulk of Sm_2CO_5 shows a layered structure, and the $Sm_2O_2^{2+}$ and CO_3^{2-} layers are parallel to the {001} plane. Three possible models of the exposed {001} plane are drawn in **Figure S1a**. In consideration of the charge separation, the section position, which locates in the interface of $Sm_2O_2^{2+}$ and CO_3^{2-} layers, shown as **Figure S1c** is unstable. For another case, the CO_3^{2-} anions are equally into two parts, shown as **Figure S1d**, and this model was still unstable for the reducing coordination numbers of both Sm and CO_3^{2-} . In the rest model, **Figure S1b**, only part of Sm—O bonds is broken, and it has a minor impact on stability. In other words, the coordination numbers of Sm and O changed from 8 and 4 to 7 and 3. So the model shown as **Figure S1b** is a more likely fracture position, and this model is also called raw surface, shown as **Figure S2a**. This name means it would be transformed into other

more stable surfaces, such as model **Figure S2b,c**. The raw surface would release 7.989 eV energy, once 6 water molecules adsorbed on it. But the most stable surface is found to be that 6 hydrolyzed Sm_2CO_5 molecules adsorbed on surface, i.e. 12 SmOOH molecules shown as **Figure S2d**. The final composite catalyst model is settled on the basis of these stable {001} surface. The detailed explanation of catalyst model is replacing three adjacent H atoms by Cu cluster, shown as **Figure S2e** and **Table S1**.

The calculated energies of different species were listed in the **Table S2– S5**.

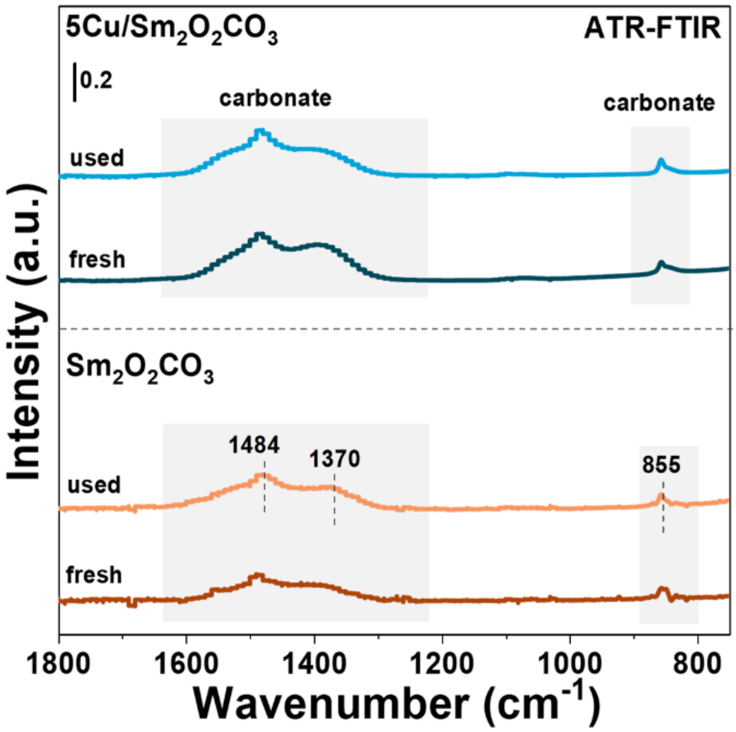

Figure S3. The ATR-FTIR spectra of the fresh and used $Sm_2O_2CO_3$ support and $5Cu/Sm_2O_2CO_3$ catalysts.

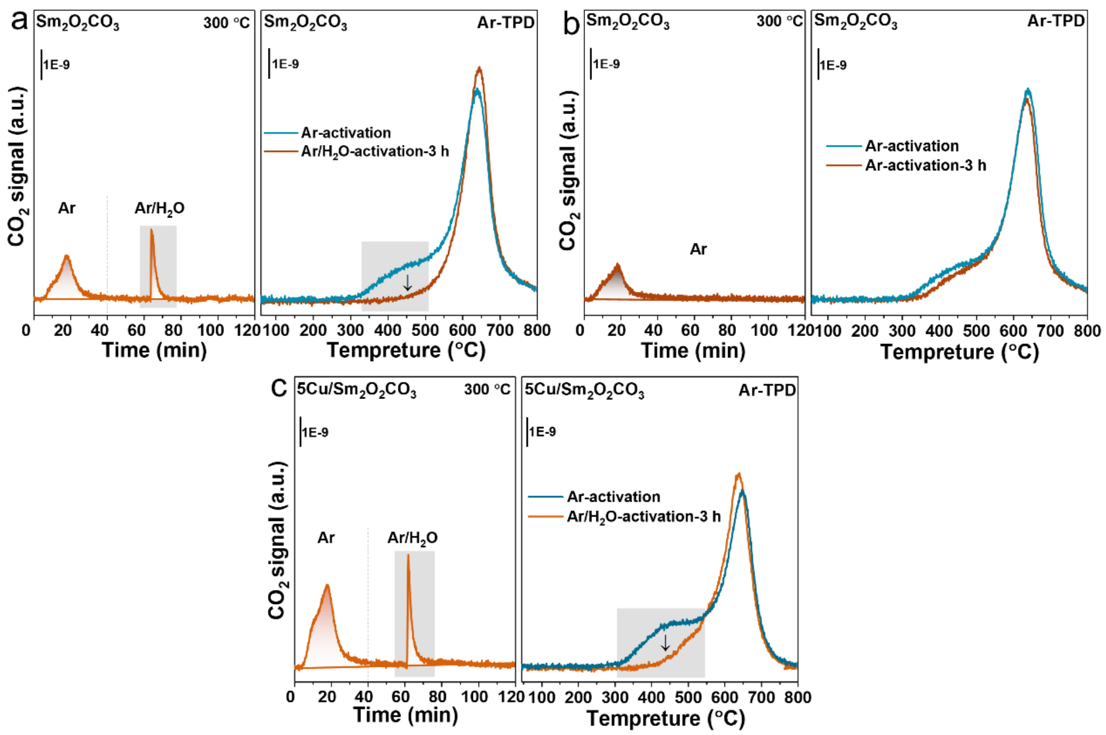

Figure S4. MS signal of CO_2 during temperature programming under Ar with and without H_2O treatment over (a, b) $Sm_2O_2CO_3$ and (c) $5Cu/Sm_2O_2CO_3$ sample.

As shown in **Figure S4a**, during the Ar treatment at 300 °C, the signal of CO_2 is generated from partial carbonate decomposition of $Sm_2O_2CO_3$, and then the gas signal disappears and remains stable, while when H_2O is introduced, the CO_2 signal suddenly increases and then decreases significantly in 10 minutes. After these operations, in the process of heating in Ar atmosphere, the CO_2 desorption peak obviously disappeared in the lower temperature region compared with the result of totally without H_2O treatment. The phenomenon strongly confirmed the displacement of carbonate species by OH generated from the decomposition of H_2O. In contrast, in the sample of $Sm_2O_2CO_3$ pretreated by converting H_2O to Ar (**Figure S4b**), there is neither a sudden increase of CO_2 signal nor the peak at low temperature still exists in the heating process under Ar gas. $5Cu/Sm_2O_2CO_3$ sample exhibits the same results as $Sm_2O_2CO_3$ shown in **Figure S4c**.

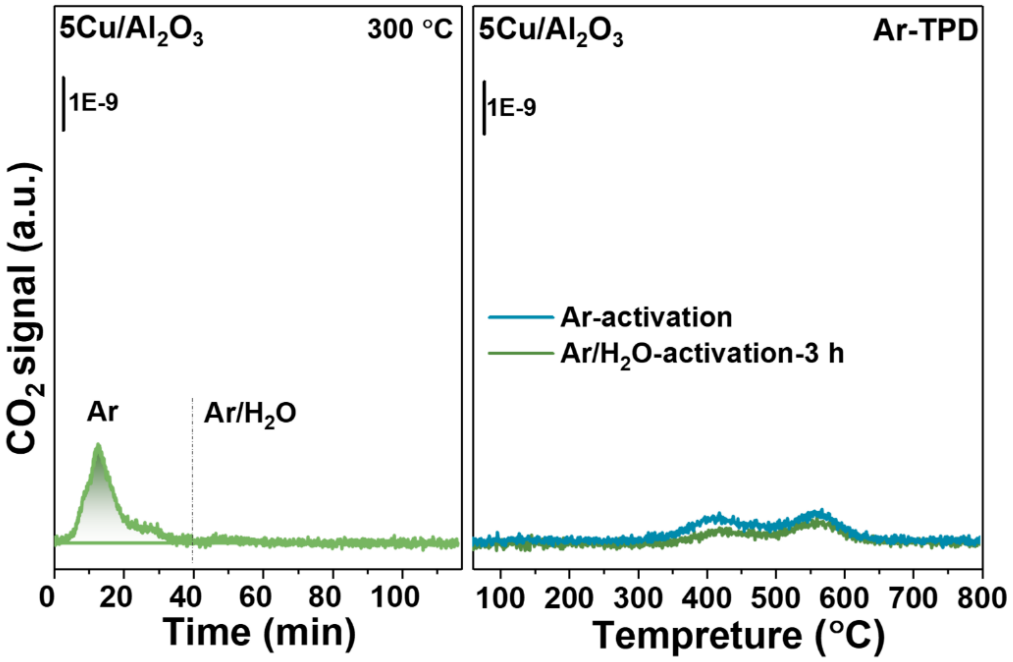

Figure S5. Ar-TPD profiles of the $5Cu/Al_2O_3$ catalyst with and without H_2O treatment.

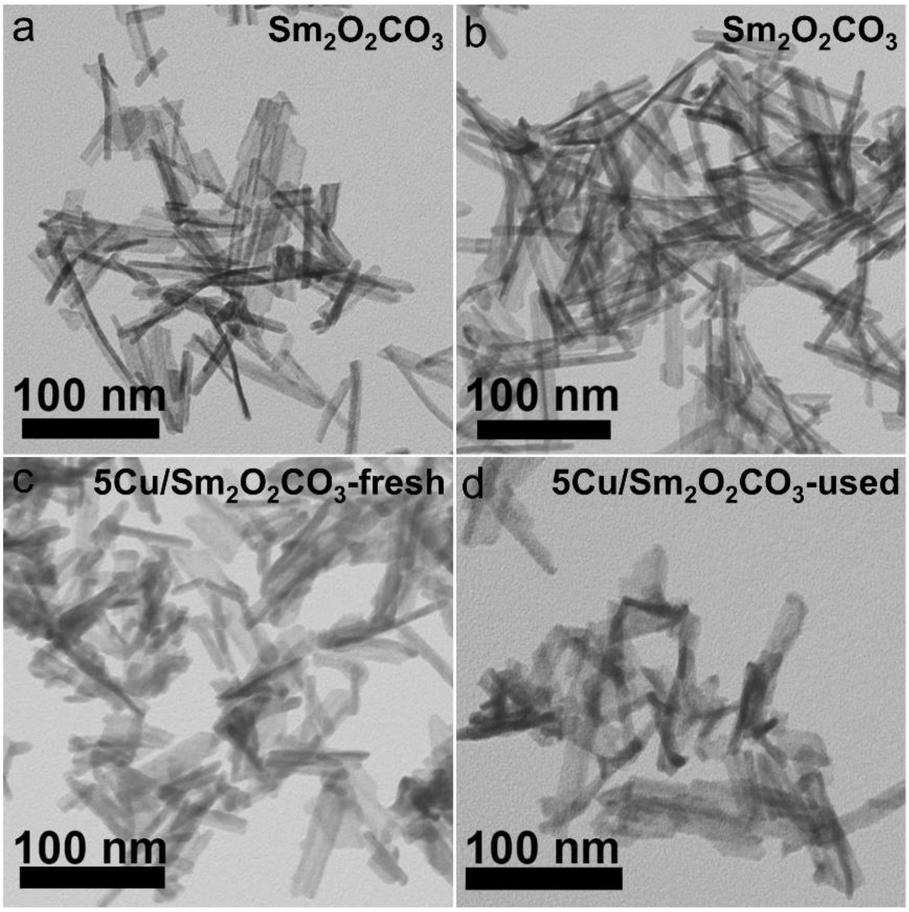

Figure S6. TEM images over the (a, b) $Sm_2O_2CO_3$, (c) $5Cu/Sm_2O_2CO_3$-fresh and (d) $5Cu/Sm_2O_2CO_3$-used catalysts.

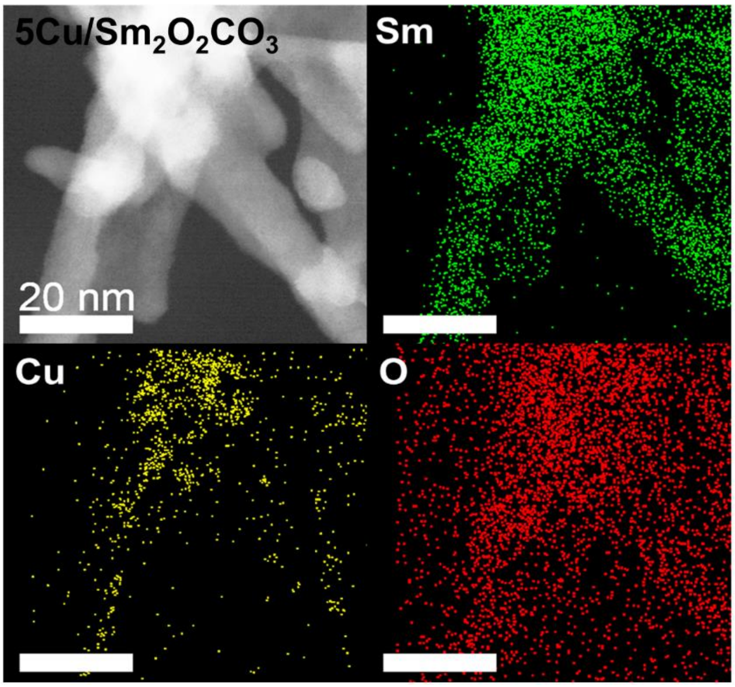

Figure S7. The EDS elemental mapping images over the 5Cu/$Sm_2O_2CO_3$ catalyst.

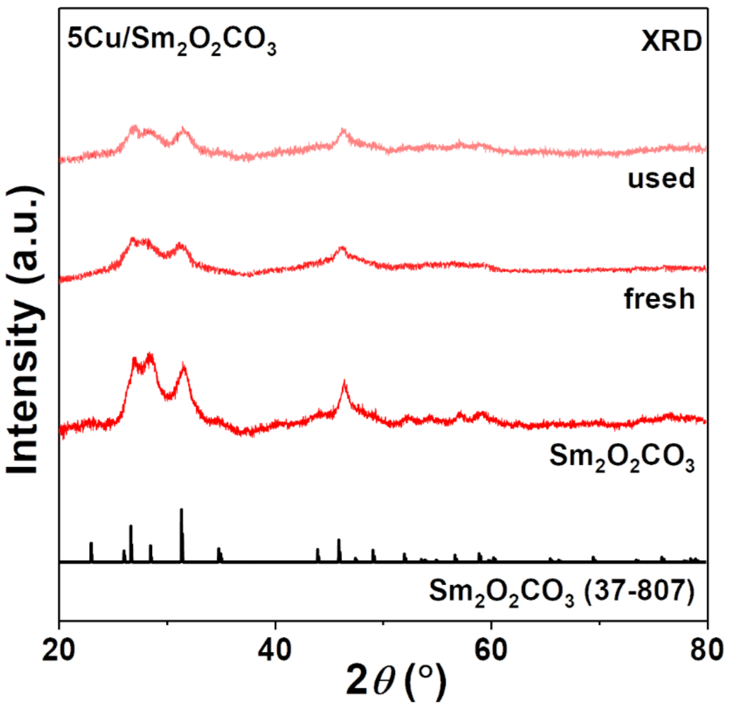

Figure S8. The XRD patterns over the $Sm_2O_2CO_3$, $5Cu/Sm_2O_2CO_3$-fresh and $5Cu/Sm_2O_2CO_3$-used catalysts.

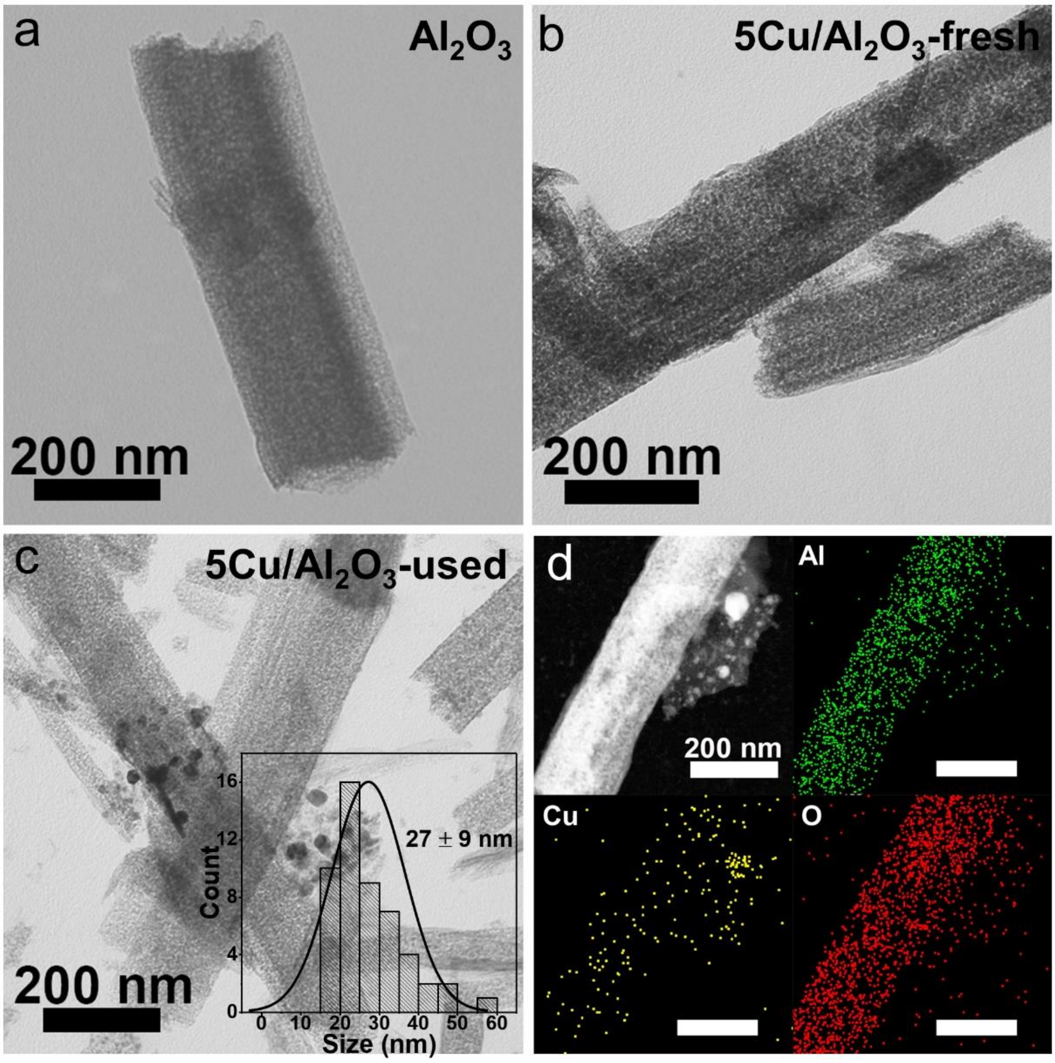

Figure S9. The TEM and EDS elemental mapping images over the Al_2O_3-based catalysts.

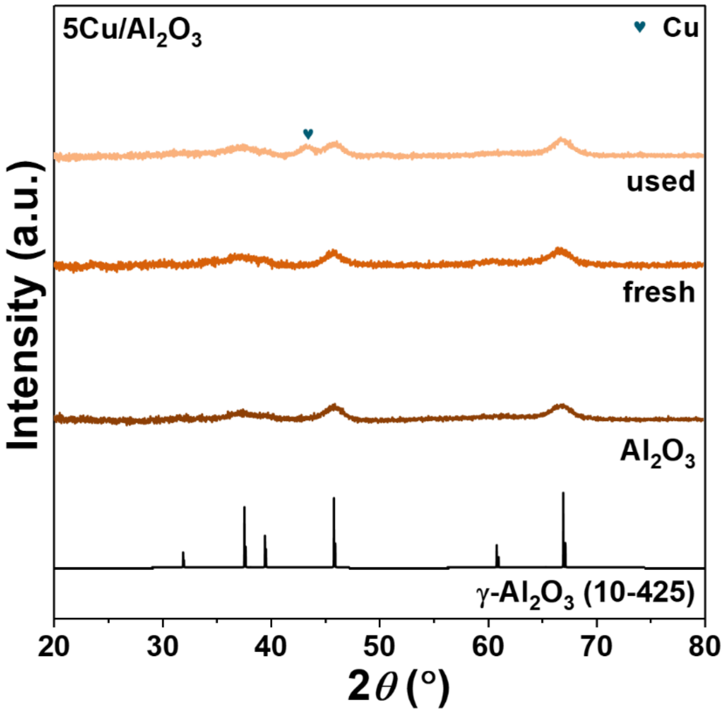

Figure S10. The XRD patterns over the Al_2O_3, 5Cu/Al_2O_3-fresh and 5Cu/Al_2O_3-used catalysts.

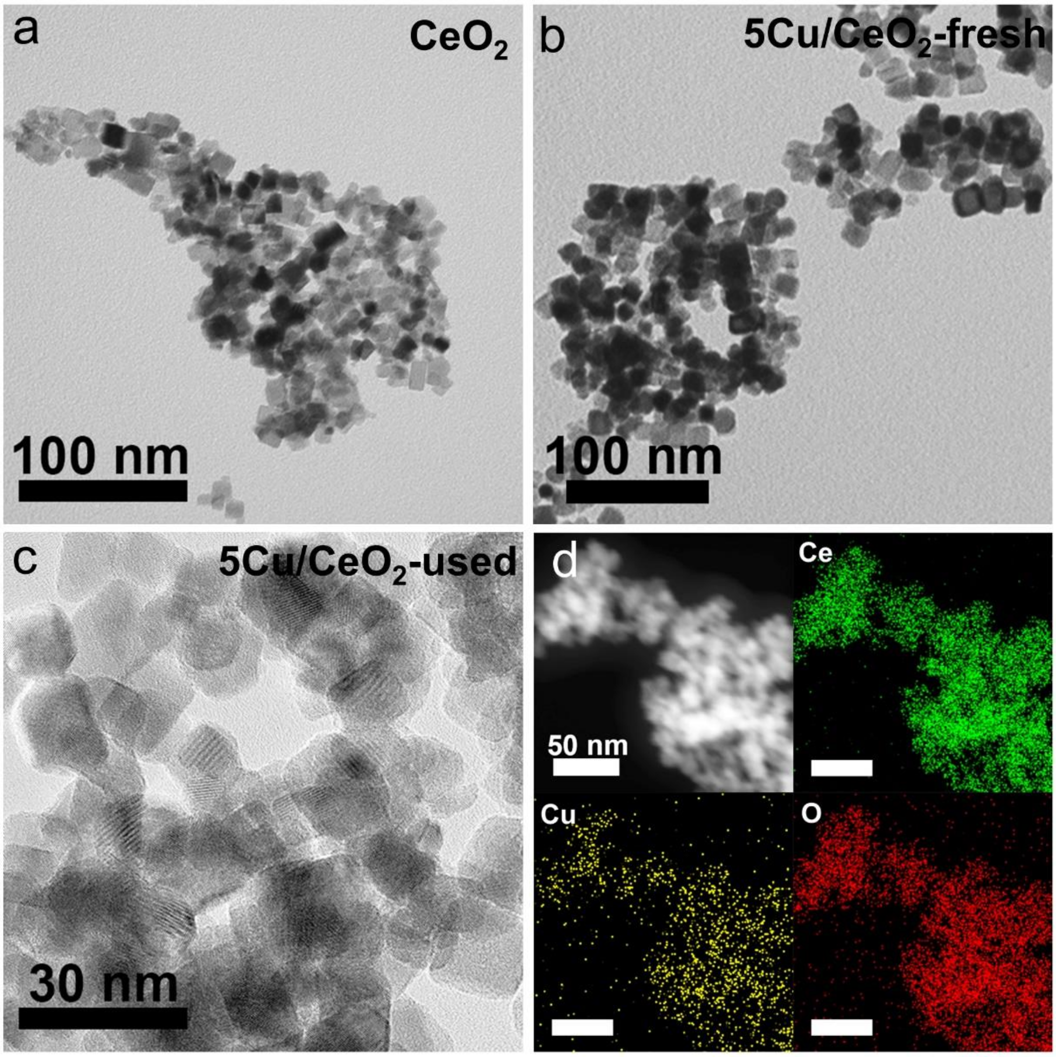

Figure S11. TEM and EDS elemental mapping images over the CeO_2-based catalysts.

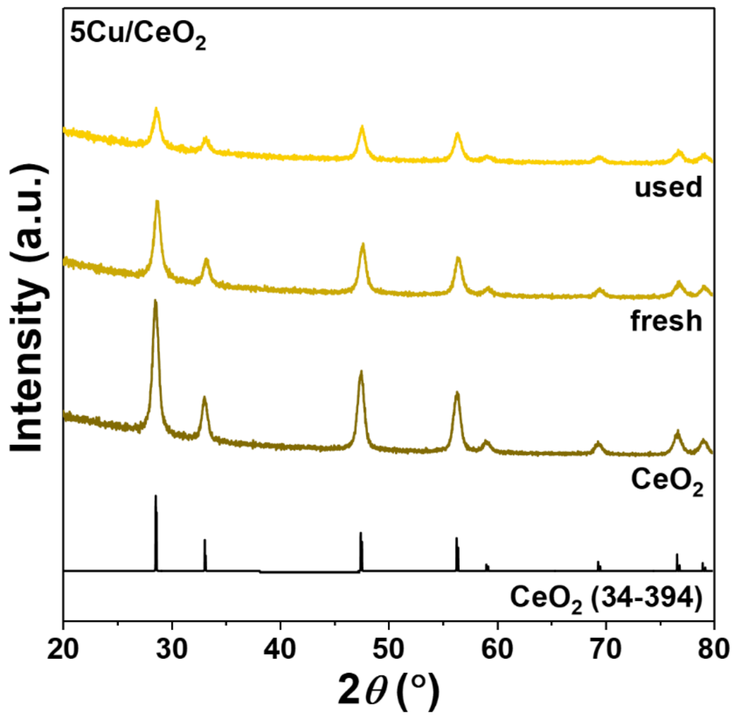

Figure S12. The XRD patterns over the CeO_2, 5Cu/CeO_2-fresh and 5Cu/CeO_2-used catalysts.

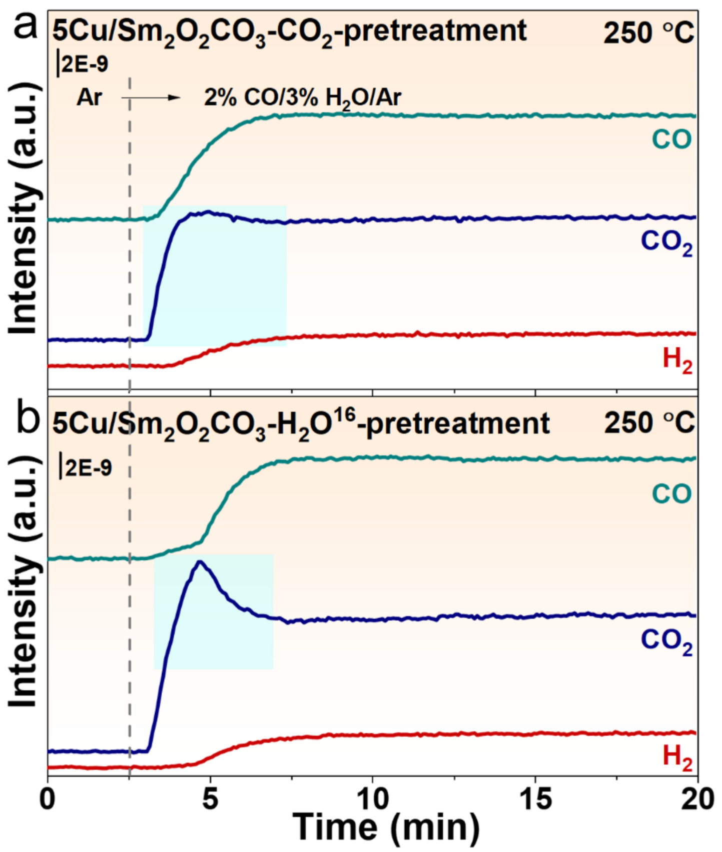

Figure S13. The signal of CO, CO_2 and H_2 during the reaction atmosphere (2% CO/3% H_2O/Ar) over the 5Cu/$Sm_2O_2CO_3$ catalyst after pretreatment with (a) 2% CO_2/Ar and (b) 10% H_2O^{16}/Ar atmosphere at 250 °C for 30 min.

The differentiated pretreatments in CO_2 or H_2O implied the CO_2-related species or H_2O-related surface species is predominant at the initial stage before gas-switching. As shown in **Figure S13a**, when the reaction gas of 2% CO/3% H_2O/Ar is introduced after the 5Cu/$Sm_2O_2CO_3$ sample pretreatment with CO_2 and purging with Ar, the CO_2 signal increases significantly within 1 min and then remains stable. While, for the sample pretreated with H_2O (**Figure S13b**), the CO_2 signal increases sharply in the beginning and then drops before remaining stable. This experimental evidence strongly indicates that the pre-exchanged H_2O molecules directly contributed to the generation of CO_2 via surface reaction.

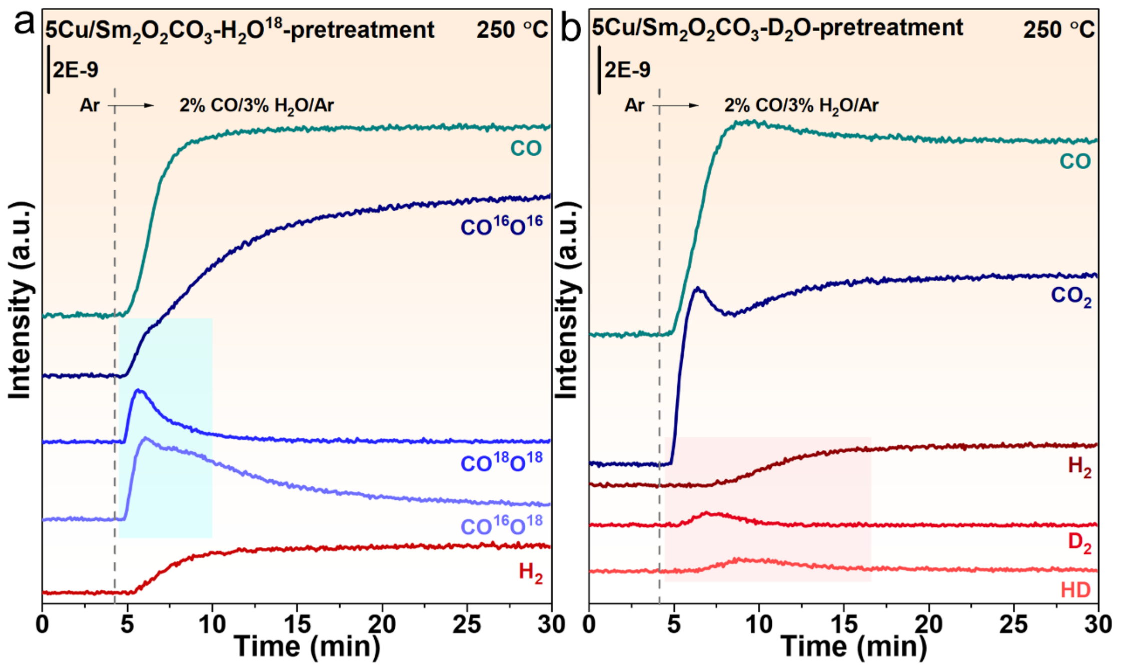

Figure S14. The signal of CO, CO_2 and H_2 during the reaction atmosphere (2% CO/3% H_2O/Ar) over the 5Cu/$Sm_2O_2CO_3$ catalyst after pretreatment with (a) 10% H_2O^{18}/Ar and (b) 10% D_2O/Ar atmosphere at 250 °C for 30 min.

In order to further clarify that the pre-exchanged process between CO_2 and H_2O is the inevitable path for the WGS reaction, we labeled the O and H atoms in H_2O molecules by using H_2O^{18} and D_2O respectively. The sample is firstly pretreated with isotopic gas of 10% H_2O^{18}/Ar or 10% D_2O/Ar and subsequently introducing the raw-gas (2% CO/3% H_2O^{16}/Ar) inside. As shown in **Figure S14a**, the signal intensity of $CO^{16}O^{18}$ and $CO^{18}O^{18}$ increases significantly, while the intensity of $CO^{16}O^{16}$ slowly increases. With the continuous inflow of reaction gas, $CO^{16}O^{18}$ and $CO^{18}O^{18}$ signals gradually decrease until disappear, while $CO^{16}O^{16}$ signals continue to increase. This confirms that the CO reacts with $*O^{18}H$ (from the dissociation of H_2O^{18} during pretreatment) in advance to form $CO^{16}O^{18}$, implying that the exchanged step is prior the WGS step under realistic reaction condition. The formation of $CO^{18}O^{18}$ may be due to the following reactions:

$$CO^{16} + *O^{18}H \rightarrow CO^{16}O^{18} + *H$$

$$*H + *H \rightarrow H_2$$

$$CO^{16}O^{18} + H_2 \rightarrow CO^{18} + H_2O^{16}$$

$CO^{18} + {}^*O^{18}H \rightarrow CO^{18}O^{18} + {}^*H$

Similarly, as shown in **Figure S14b**, further evidence can be derived from the D-labeled experiments. After the introduction of reaction gas, D_2 (m/z = 4) is generated first and followed by the signals of HD (m/z = 3) as well as H_2 (m/z = 2), **confirming that the CO firstly reacts with dissociated *OD groups generated during exchanging pretreatment.** The formation of HD may be due to the following reactions:

$CO + {}^*OD \rightarrow CO_2 + {}^*D$

${}^*D + {}^*D \rightarrow D_2$

$CO + {}^*OH \rightarrow CO_2 + {}^*H$

${}^*H + {}^*D \rightarrow HD$

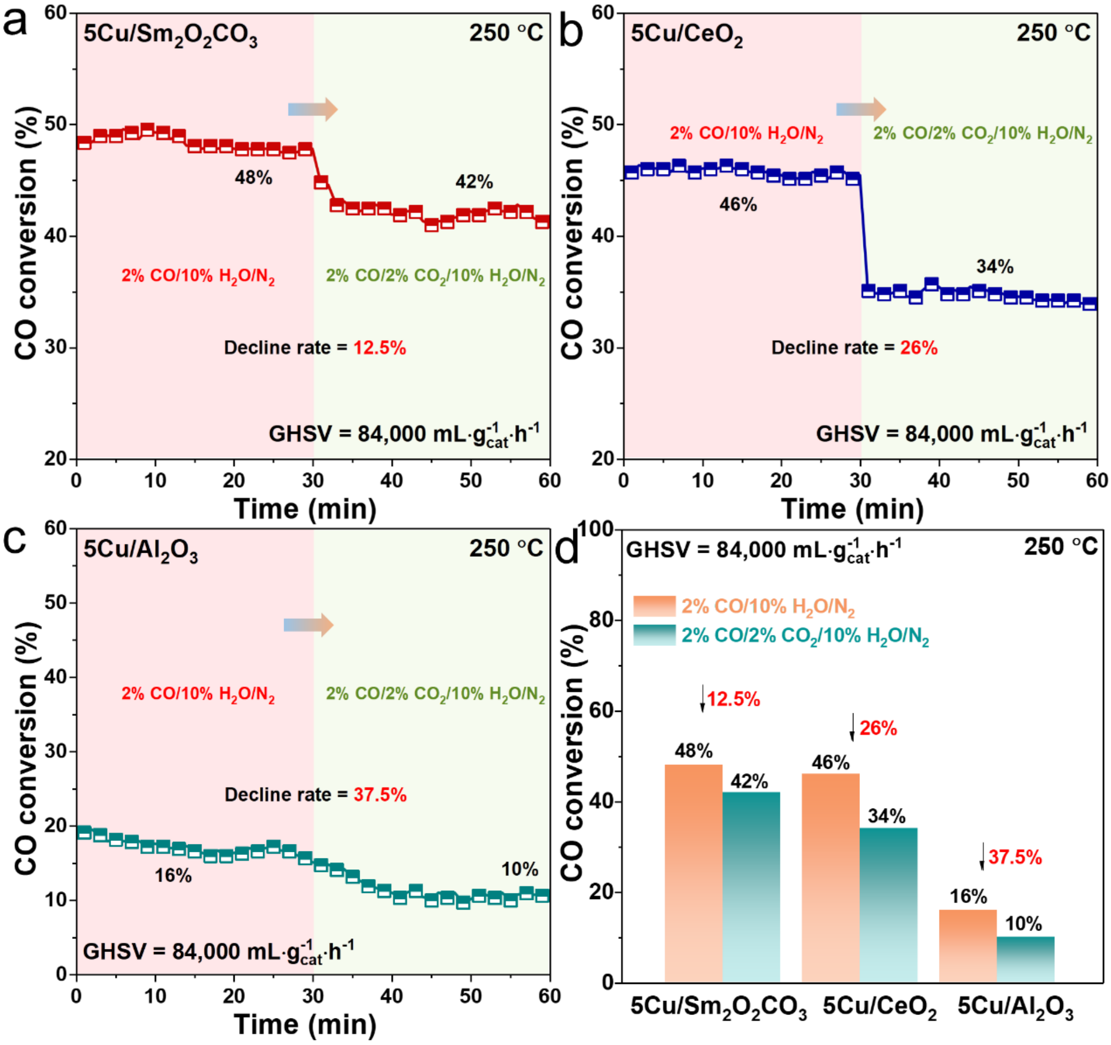

Figure S15. The catalytic performances test under different reaction atmospheres (2% CO/10% H_2O/N_2 or 2% CO/2% CO_2/10% H_2O/N_2) over (a) 5Cu/$Sm_2O_2CO_3$, (b) 5Cu/CeO_2 and (c) 5Cu/Al_2O_3 catalysts. (d) Histogram analysis of activity over three catalysts in different atmospheres.

In addition to the dynamic switching experiments with MS, the stable apparent activity data is performed to demonstrate the validity of the exchange of *OH and *CO_3. As shown in **Figure S15**, after switching the mixture of 2% CO/10% H_2O/N_2 to 2% CO/2%CO_2/10% H_2O/N_2, the decline rates of CO conversion rate are 12.5%, 26% and 37.5% with the space velocity of 84,000 mL·g^{-1}·h^{-1} for 5Cu/$Sm_2O_2CO_3$, 5Cu/CeO_2 and 5Cu/Al_2O_3 catalysts, respectively. The above results clearly indicate that the 5Cu/$Sm_2O_2CO_3$ catalyst possesses the lowest decline rates of CO conversion rate compared to the other two samples. **This corresponds to the exchange capacity of *OH and *CO_3 on the surface of 5Cu/$Sm_2O_2CO_3$ catalyst, and the *OH can**

effectively replace the carbonate species formed by CO_2 adsorption and accelerate the reaction process.

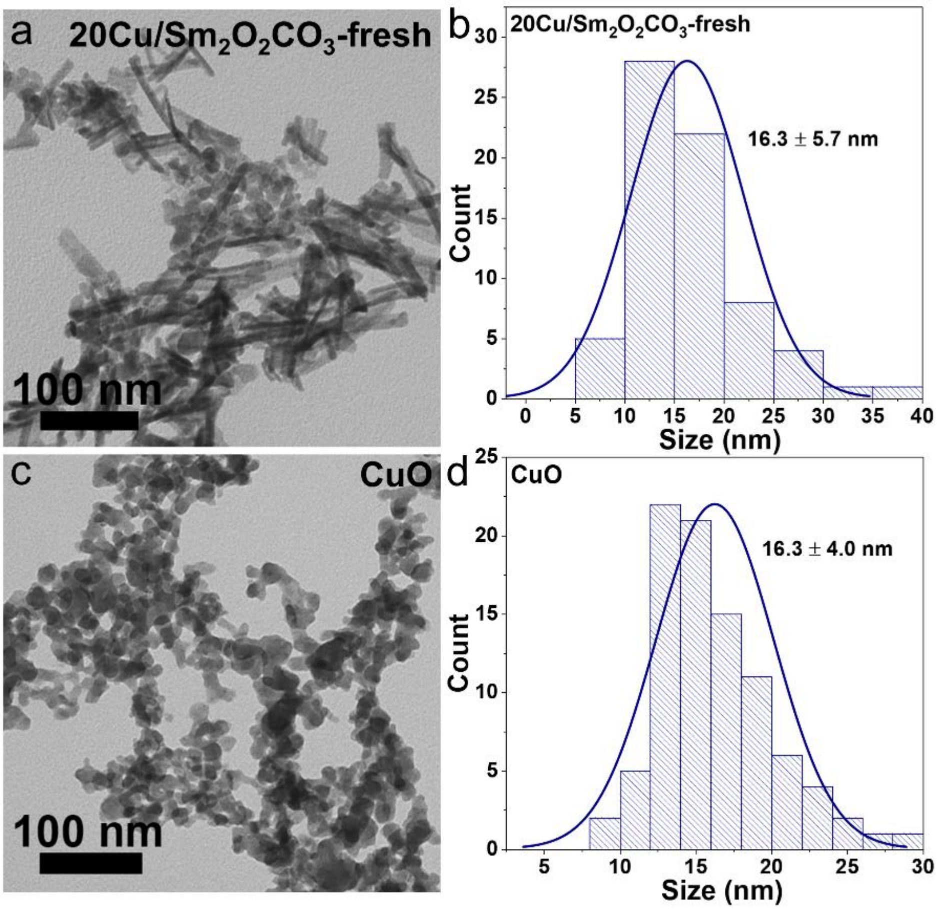

Figure S16. The TEM images and particle size statistics of Cu species over catalysts: (a, b) 20Cu/$Sm_2O_2CO_3$-fresh and (c, d) CuO catalysts.

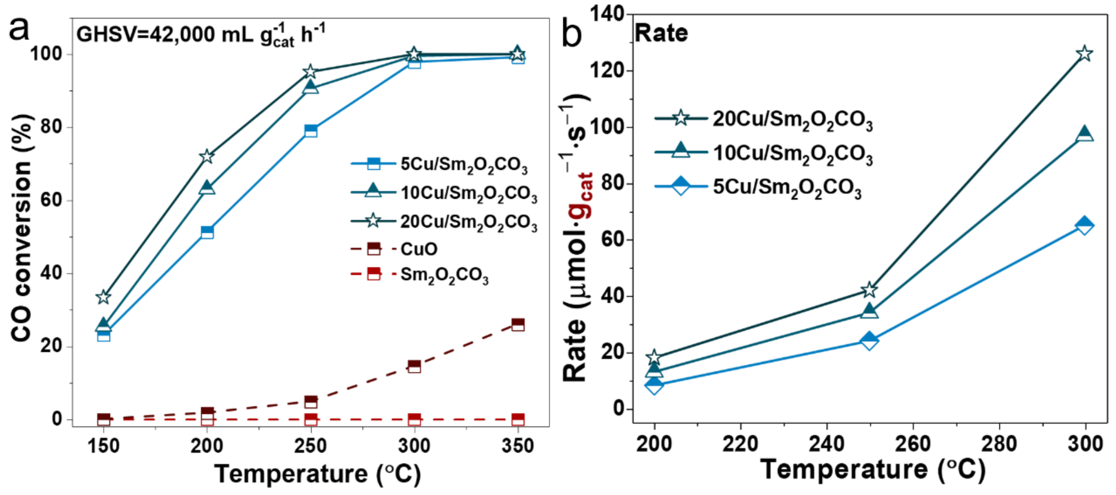

Figure S17. (a) The water–gas shift (WGS) reaction activities of the *x*Cu/$Sm_2O_2CO_3$ catalysts, pure CuO and $Sm_2O_2CO_3$ support; (b) The reaction rates normalized by catalyst weight at various temperatures for *x*Cu/ $Sm_2O_2CO_3$ catalysts.

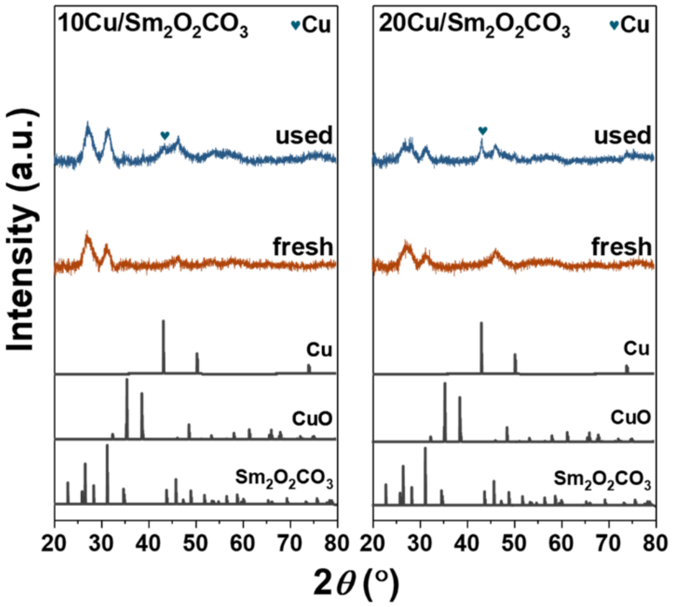

Figure S18. The XRD patterns of the fresh and used 10Cu/$Sm_2O_2CO_3$ and 20Cu/$Sm_2O_2CO_3$ catalysts.

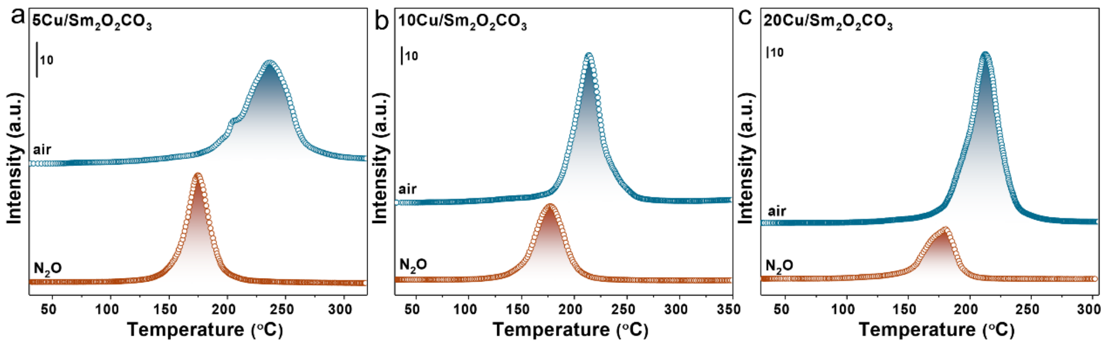

Figure S19. The H_2-TPR profiles over various Cu/ $Sm_2O_2CO_3$ catalysts under air and N_2O pretreatment.

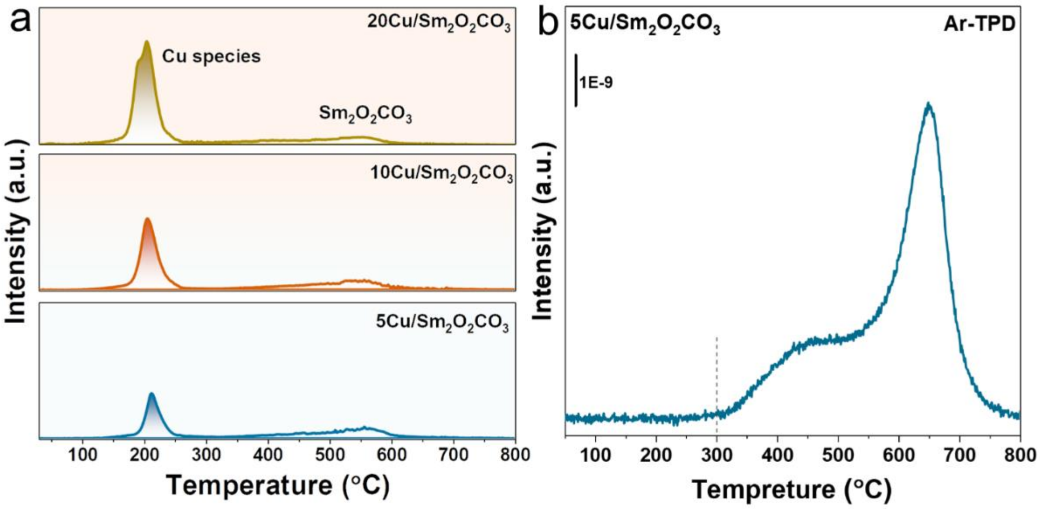

Figure S20. (a) The H_2-TPR profiles over various Cu/$Sm_2O_2CO_3$ catalysts and (b) MS signal of CO_2 of 5Cu/$Sm_2O_2CO_3$ catalyst during temperature programming under Ar.

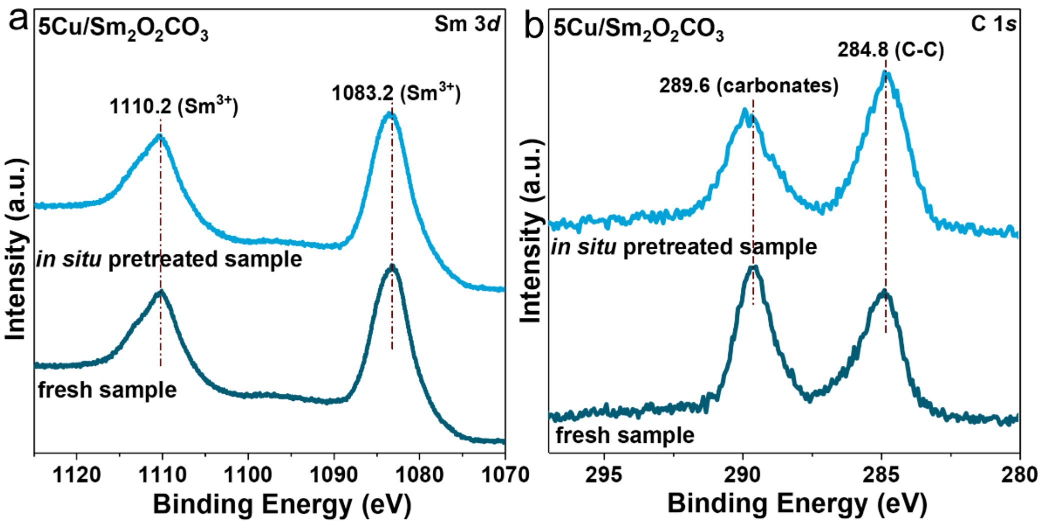

Figure S21. *Ex-situ* and quasi *in-situ* Sm 3*d* and C 1*s* XPS spectra of fresh and spent $5Cu/Sm_2O_2CO_3$ catalyst.

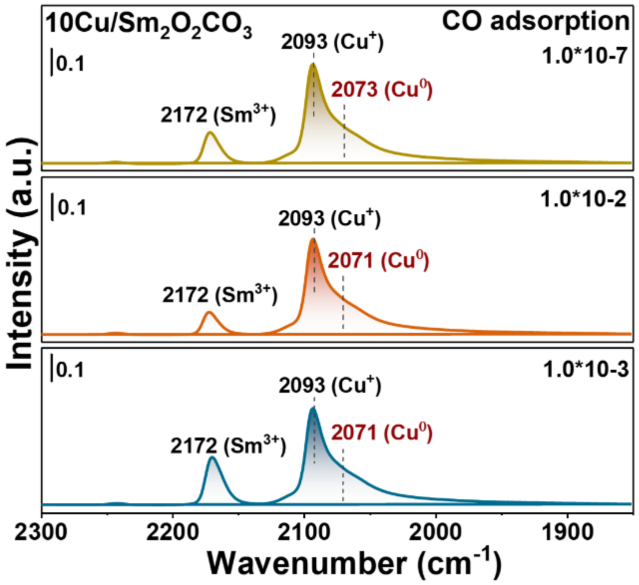

Figure S22. *In-situ* infrared spectra of the CO adsorption for 10Cu/$Sm_2O_2CO_3$ catalyst under different CO pressures (1.0×10^{-3} and 1.0×10^{-2} mbar) and vacuum conditions (1.0×10^{-7} mbar).

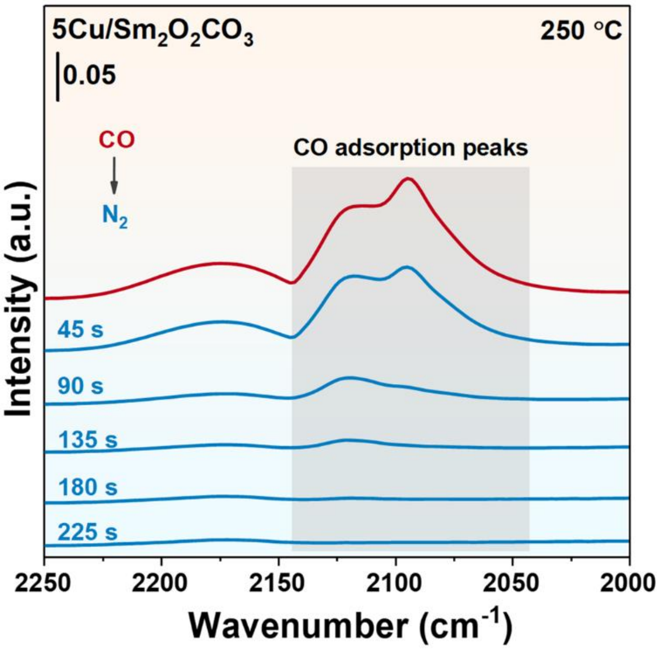

Figure S23. *In-situ* DRIFTS spectra of CO adsorption and N_2 purging over $5Cu/Sm_2O_2CO_3$ catalyst at 250 °C.

As shown in **Figure S23**, the intensity of CO chemisorbed on Cu species decreases significantly within 90 s and almost completely disappears after 225 s, indicating the weak bonding ability of CO to metal.

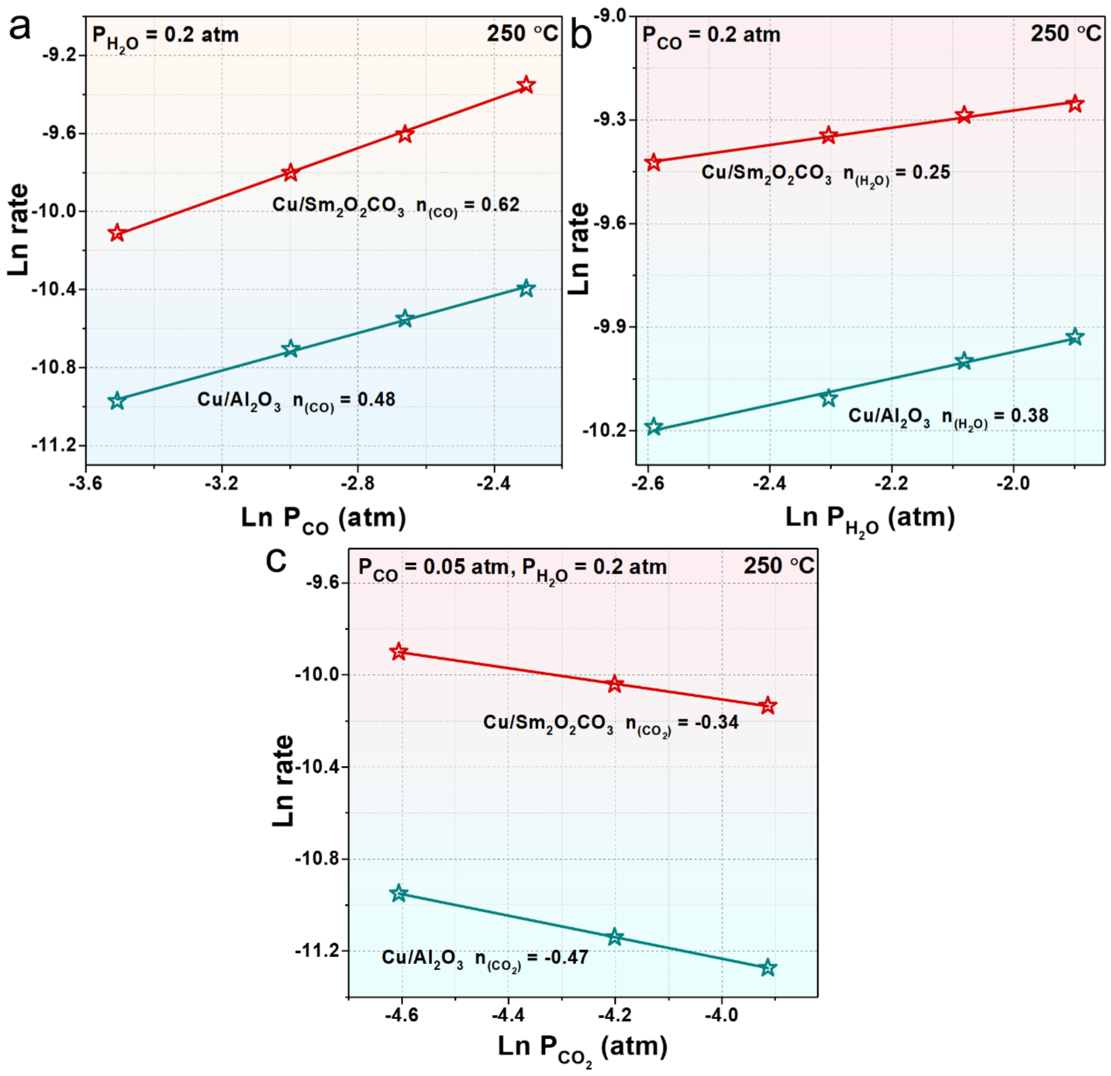

Figure S24. Kinetic orders of (a) CO, (b) H_2O and (c)CO_2 for $5Cu/Sm_2O_2CO_3$ and $5Cu/Al_2O_3$ catalysts at 250 °C, respectively.

For the kinetic orders test, the reactants CO, H_2O and the product CO_2 are measured for $5Cu/Sm_2O_2CO_3$ and $5Cu/Al_2O_3$ these two catalysts at 250 °C. As shown in **Figure S24a**, upon further increase of the CO partial pressure, the reaction rates of catalysts are gradually increasing, and the $5Cu/Sm_2O_2CO_3$ catalyst exhibits the higher value of CO reaction order compared to $5Cu/Al_2O_3$ sample, demonstrating that the CO is not saturated on the $5Cu/Sm_2O_2CO_3$ catalyst surface and will not cover the active sites. In the kinetic orders test of H_2O and CO_2 (**Figure S24b,c**), with the increasement of H_2O and CO_2 partial pressure, the reaction rates increase and decrease, respectively. In addition, compared with the $5Cu/Al_2O_3$ catalyst, the $5Cu/Sm_2O_2CO_3$ gives lower absolute value of kinetic orders with 0.34 (*vs.* 0.47) and 0.25 (*vs.* 0.38) for CO_2 and

H_2O, respectively, proving the weaker negative effect from the product CO_2 and stronger positive effect from the dissociation of the reactant H_2O. This is related to the effective exchange of $*CO_3$ and $*OH$ on the $5Cu/Sm_2O_2CO_3$ catalyst surface to promote H_2O dissociation and CO_2 desorption and accelerate the WGS reaction process.

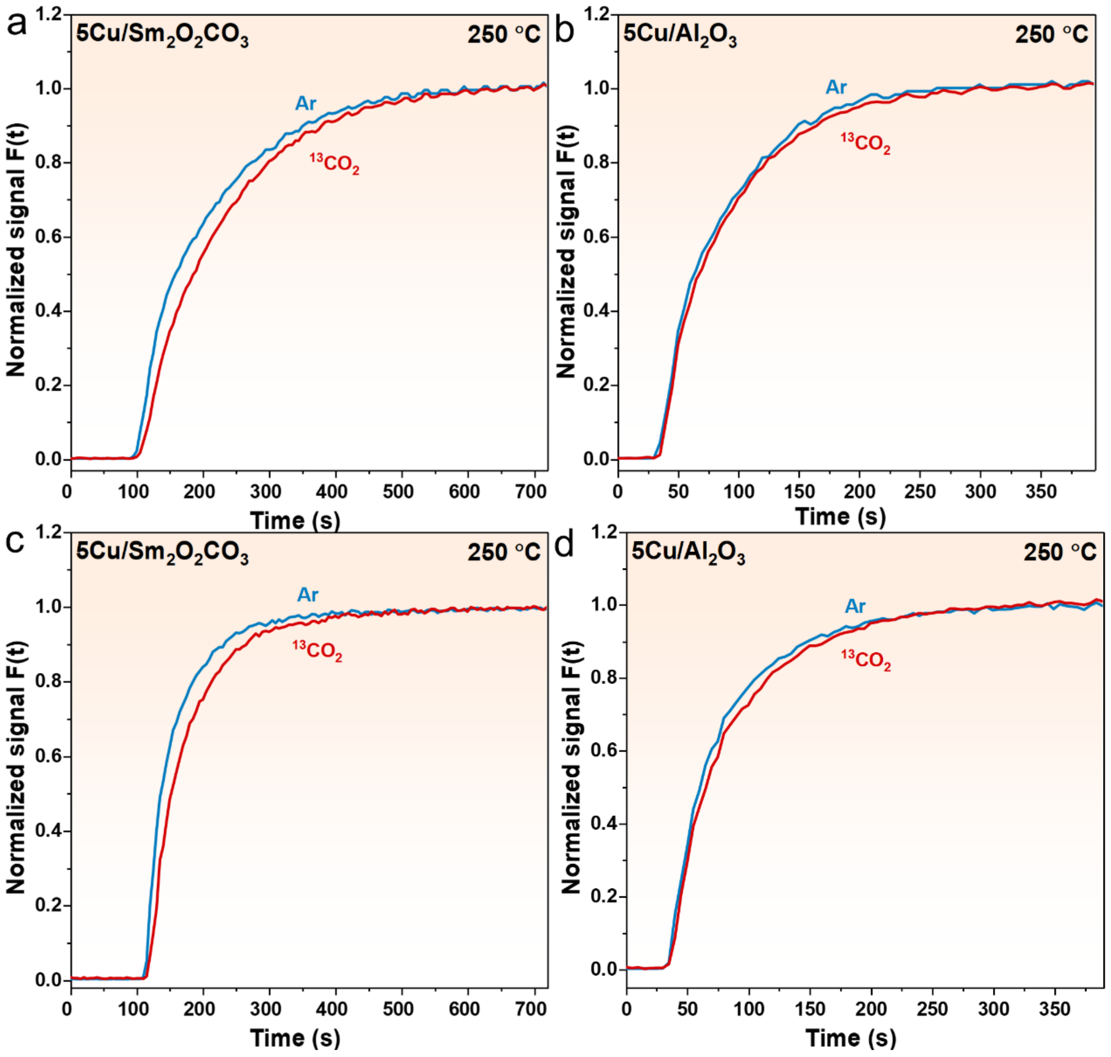

Figure S25. The steady-state isotopic transients of 5% $^{12}CO_2$/He to 5% $^{13}CO_2$/Ar for (a) 5Cu/$Sm_2O_2CO_3$ and (b) 5Cu/Al_2O_3 catalysts and the steady-state isotopic transients of 5% $^{12}CO_2$/10% H_2O/He to 5% $^{13}CO_2$/10% H_2O/Ar for (c) 5Cu/$Sm_2O_2CO_3$ and (d) 5Cu/Al_2O_3 catalysts at 250 °C.

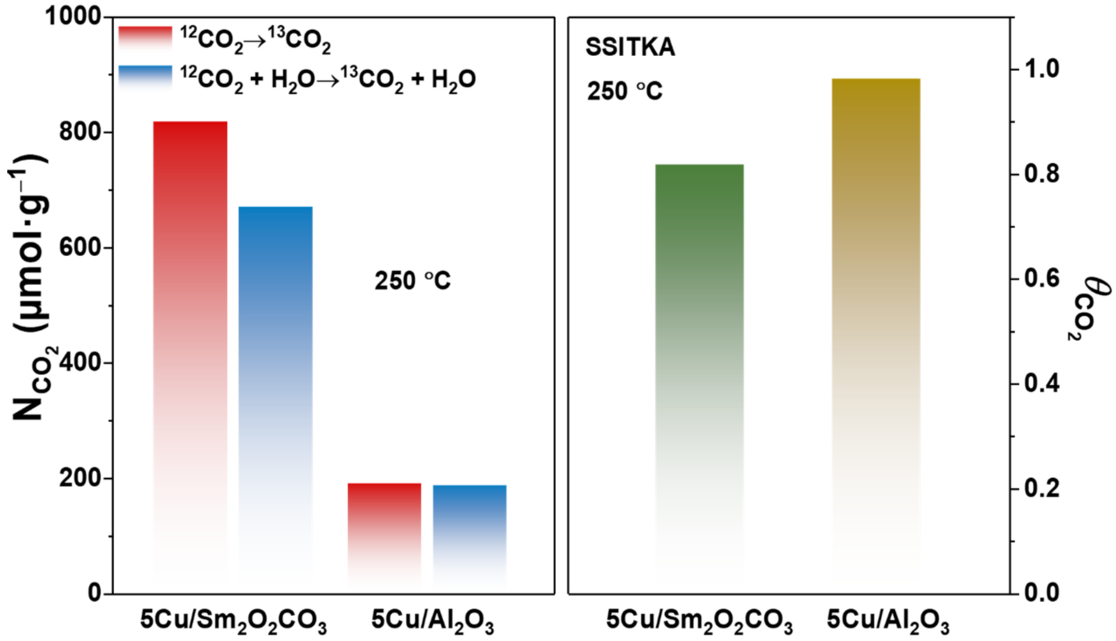

Figure S26. The concentration and surface coverage of CO_2 in the SSITKA–MS studies at 250 °C for 5Cu/$Sm_2O_2CO_3$ and 5Cu/Al_2O_3 catalysts.

As shown in **Figure S25 and S26**, the normalized signals of isotope-labelled $^{13}CO_2$ and Ar are recorded during the switching of 5% $^{12}CO_2$/He to 5% $^{13}CO_2$/Ar (**Figure S25a and b**) or 5% $^{12}CO_2$/10% H_2O/He to 5% $^{13}CO_2$/10% H_2O/Ar (**Figure S25c,d**) for 5Cu/$Sm_2O_2CO_3$ and 5Cu/Al_2O_3 catalysts at 250 °C. The results of the SSITKA analysis (**Figure S26**) show that the 5Cu/$Sm_2O_2CO_3$ sample demonstrated a lower θ_{CO_2} of 0.82 than that of 5Cu/Al_2O_3 catalyst (θ_{CO_2} = 0.98), demonstrating that the H_2O molecules are relatively apt to replace generated CO_2 molecules under reaction condition.

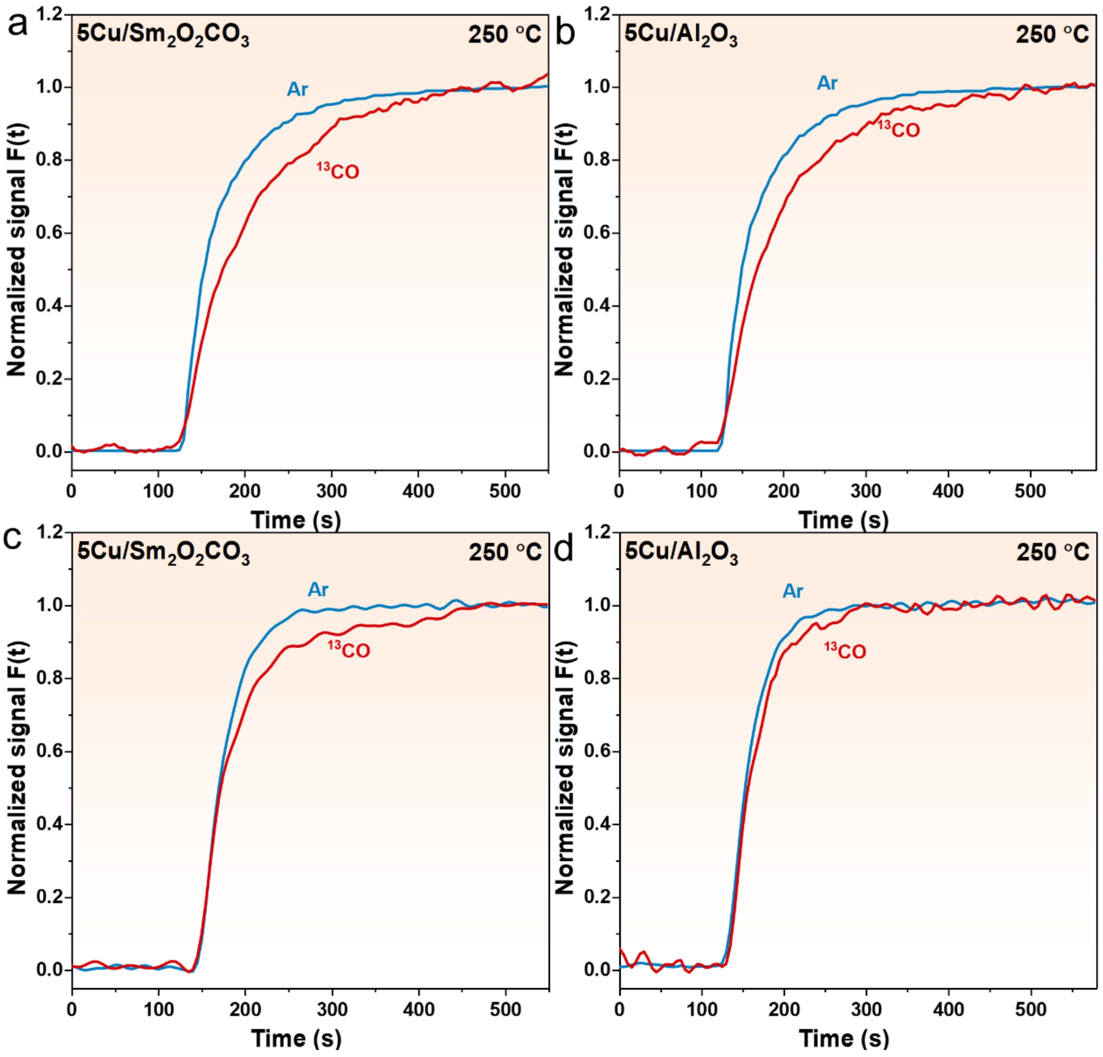

Figure S27. The steady-state isotopic transients of 0.5% ^{12}CO/He to 0.5% ^{13}CO/Ar for (a) 5Cu/$Sm_2O_2CO_3$ and (b) 5Cu/Al_2O_3 catalysts and the steady-state isotopic transients of 0.25% ^{12}CO/1% CO_2/50% Ar/He to 0.25% ^{13}CO/1% CO_2/Ar for (a) 5Cu/$Sm_2O_2CO_3$ and (b) 5Cu/Al_2O_3 catalysts at 250 °C.

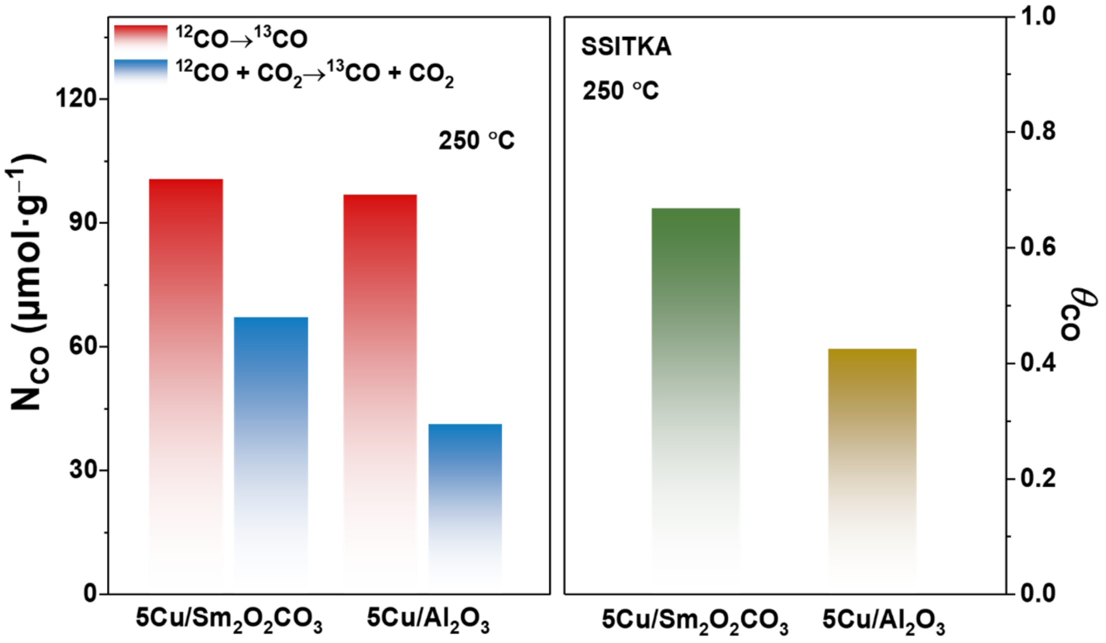

Figure S28. The concentration and surface coverage of CO in the SSITKA–MS studies at 250 °C for 5Cu/$Sm_2O_2CO_3$ and 5Cu/Al_2O_3 catalysts.

Similarly, by *in-situ* switching ^{12}CO/He to ^{13}CO/Ar with or without mixture of 1% CO_2/Ar inside, the total number of active sites (N_{tot}) and adsorbed amount of reversibly adsorbed CO (N_{CO}) molecules under steady state are determined. Subsequently, dividing the N_{CO} with N_{tot} gives the surface coverage of CO (θ_{CO}) with the presence of CO_2 molecules. As shown in **Figure S27**, the normalized signals of isotope-labelled ^{13}CO and Ar are collected during the switching of 0.5% ^{12}CO/He to 0.5% ^{13}CO/Ar (**Figure S27a,b**) or 0.25% ^{12}CO/1% CO_2/50% Ar/He to 0.25% ^{13}CO/1% CO_2/Ar (**Figure S27c,d**) for 5Cu/$Sm_2O_2CO_3$ and 5Cu/Al_2O_3 catalysts at 250 °C. The results of the SSITKA analysis (**Figure S28**) show that the 5Cu/$Sm_2O_2CO_3$ sample shows the higher value of CO surface coverage to 5Cu/Al_2O_3 (0.67 *vs.* 0.42), indicating the lower effect of product CO_2 on the adsorption of the reactant CO. This further indicates the importance of *OH and *CO_3 exchange to facilitate CO adsorption and further accelerated the WGS reaction process.

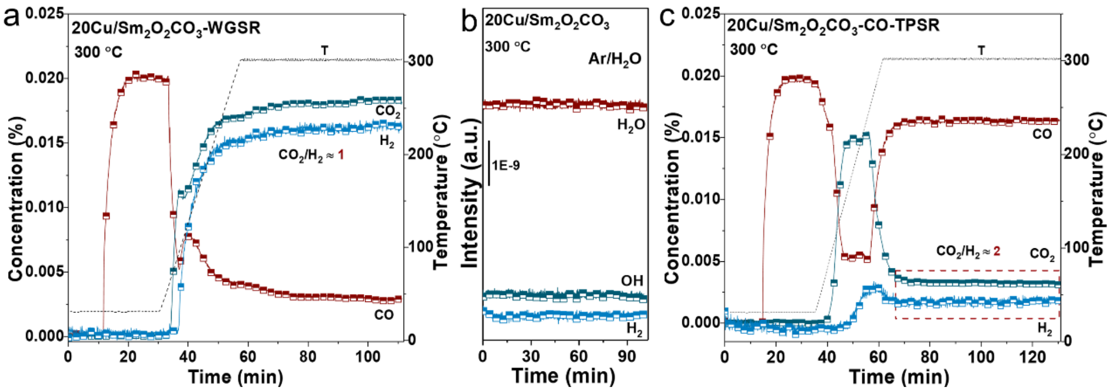

Figure S29. WGS reaction mechanism study of the 20Cu/$Sm_2O_2CO_3$ catalyst. (a) *In-situ* WGS reaction at 300 °C; (b) the experiment of H_2O dissociation; (c) temperature programmed surface reduction (TPSR) of the catalyst.

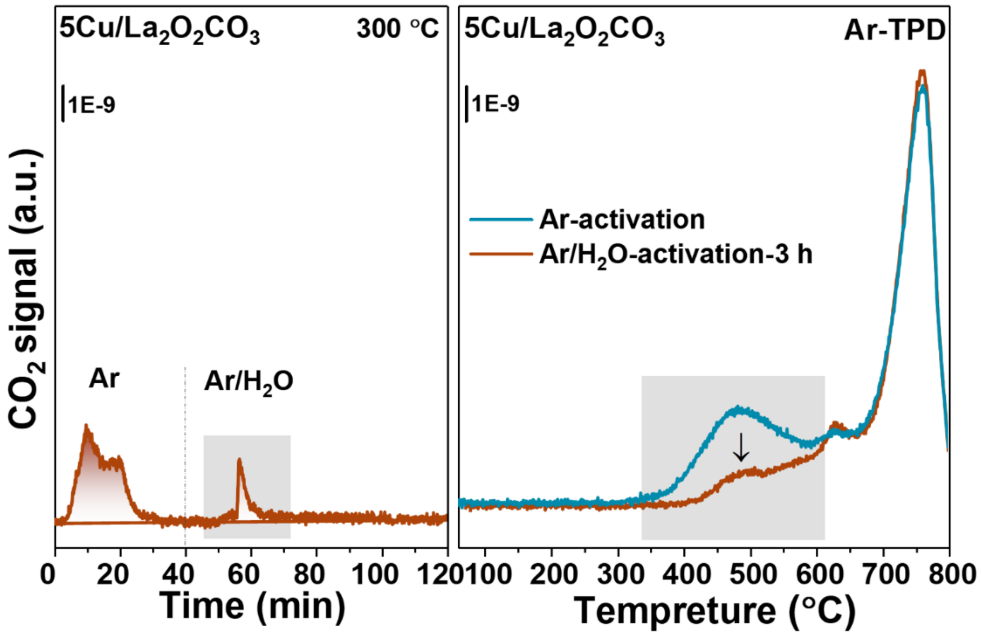

Figure S30. Ar-TPD profiles of the 5Cu/$La_2O_2CO_3$ catalysts with and without H_2O treatment.

As shown in **Figure S30**, the 5Cu/$La_2O_2CO_3$ catalyst exhibits the similar properties to Cu/$Sm_2O_2CO_3$ of the exchange capacity of CO_2 and H_2O on the surface of catalyst. After introducing of H_2O, the CO_2 signal suddenly increases, and the intensity of the peak in the low-temperature region decreases significantly during the next heating process in Ar gas compared to the sample not be treated with H_2O.

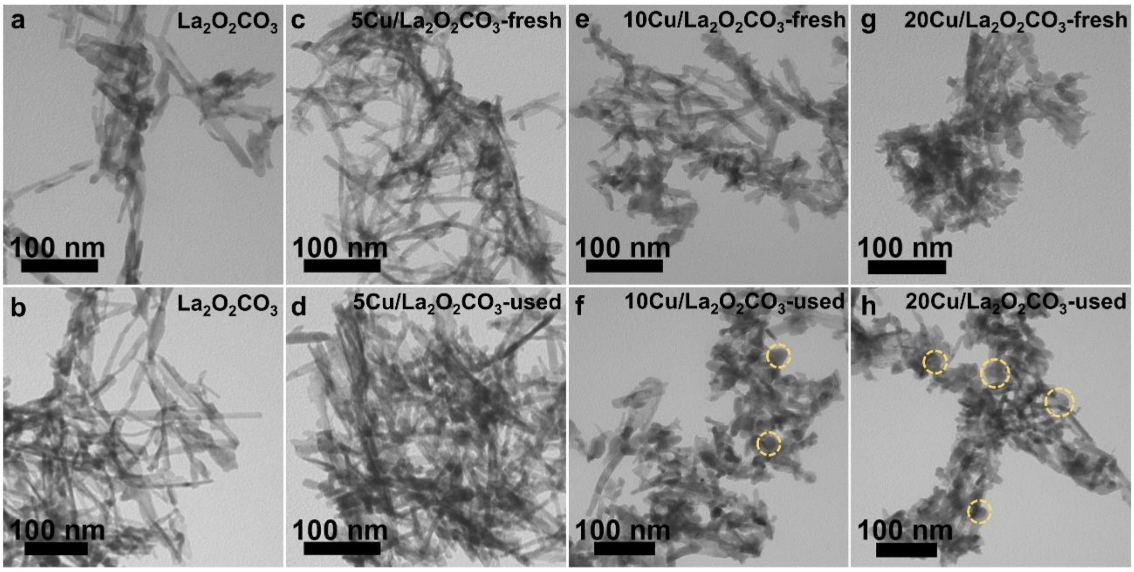

Figure S31. TEM images over the (a, b) $La_2O_2CO_3$, (c, d) $5Cu/La_2O_2CO_3$, (e, f) $10Cu/La_2O_2CO_3$ and (g, h) $20Cu/La_2O_2CO_3$ catalysts.

As shown in **Figure S31**, the support of $La_2O_2CO_3$ presents the nanorod-like morphology accompanied with slight sintering. After loading Cu species with different contents on $La_2O_2CO_3$, the samples with low Cu content have no obvious change, while there are precipitated Cu particles in the yellow circles over the samples with high Cu loading.

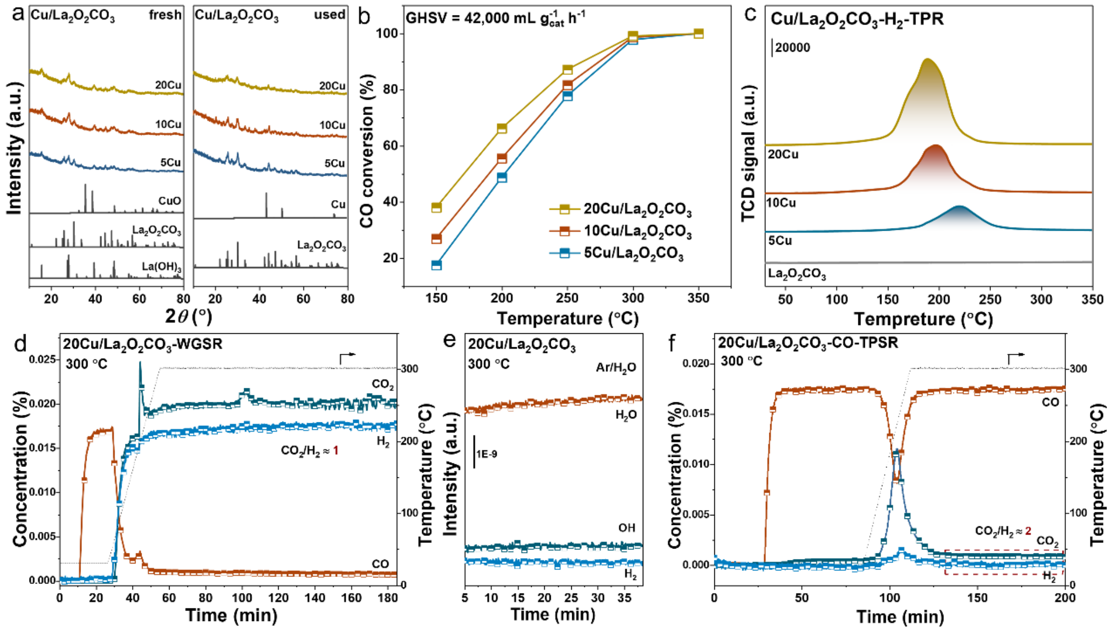

Figure S32. (a) XRD patterns over fresh and used *x*Cu/$La_2O_2CO_3$ (*x* = 5, 10 and 20) catalysts; (b) The water–gas shift (WGS) reaction activities of the *x*Cu/$La_2O_2CO_3$ (*x* = 5, 10 and 20) catalysts; (c) H_2-TPR profiles over $La_2O_2CO_3$ and *x*Cu/$La_2O_2CO_3$ (*x* = 5, 10 and 20) catalysts; (d) The concentration of each reactant molecule and product molecule during the in-situ WGS reaction at 300 °C of 20Cu/$La_2O_2CO_3$ catalyst; (e) After the in-situ WGS reaction and switching to H_2O-Ar atmosphere, the MS signals of H_2O, OH and H_2 over 20Cu/$La_2O_2CO_3$ catalyst; (f) The temperature programmed surface reduction (TPSR) of the 20Cu/$La_2O_2CO_3$ catalyst.

As shown in **Figure S32a**, the XRD patterns exhibit that the support is the mixture of $La(OH)_3$ and $La_2O_2CO_3$ before the reaction, while it exists as the separate $La_2O_2CO_3$ phase after the reaction. The absence of the characteristic diffraction peaks for Cu species may be due to their amorphous state. **Figure S32b** demonstrates the reactivity of WGS reaction over *x*Cu/$La_2O_2CO_3$ (*x* = 5, 10 and 20) catalysts. The activity increased slightly with increasing Cu content, reaching ~80–90% CO conversion at 250 °C, which similar to the *x*Cu/$Sm_2O_2CO_3$ reactivity. In **Figure S32c**, it can be seen that with the increase of Cu content, the reduction peaks shift to the lower temperature for *x*Cu/$La_2O_2CO_3$ samples, which indicates that there is a stronger interaction between Cu and the support in samples with lower Cu content. **Figure S32d–f** explores the catalytic mechanism of 20Cu/$La_2O_2CO_3$ sample for WGS reaction, during the WGS reaction (**Figure S32d**), The ratio of CO_2 to H_2 is well

maintained at ~1. No H_2 signal is observed during the introduction of H_2O (**Figure S32e**), which rules out the redox mechanism. In the following CO-TPSR test, the ratio of CO_2 and H_2 is maintained at ~2, which confirms the associative mechanism. The above-mentioned explorations on the reactivity, the interaction between Cu and support and the mechanism of Cu/$La_2O_2CO_3$ samples are consistent with the results of Cu/$Sm_2O_2CO_3$ samples, indicating that we have successfully extended the Cu/$Sm_2O_2CO_3$ system to Cu/$La_2O_2CO_3$.

Table S1. The fractional coordinates of the catalyst model. (The cell parameters: a=13.5252; b=11.7132; c=34.3707;α=β=γ=90°)

atom	a	b	c	atom	a	b	c
Sm	0.052740	0.227860	0.024844	O	0.302139	0.062614	0.232118
Sm	0.302740	0.061190	0.024844	O	0.552170	0.229214	0.232111
Sm	0.552740	0.227860	0.024844	O	0.802147	0.062559	0.232138
Sm	0.802740	0.061190	0.024844	O	0.052155	0.562613	0.232140
Sm	0.052740	0.561190	0.024844	O	0.302193	0.395937	0.232131
Sm	0.302740	0.394520	0.024844	O	0.552204	0.562534	0.232118
Sm	0.552740	0.561190	0.024844	O	0.802192	0.395867	0.232134
Sm	0.802740	0.394520	0.024844	O	0.052133	0.895933	0.232128
Sm	0.052740	0.894520	0.024844	O	0.302143	0.729287	0.232127
Sm	0.302740	0.727860	0.024844	O	0.552175	0.895907	0.232128
Sm	0.552740	0.894520	0.024844	O	0.802187	0.729177	0.232123
Sm	0.802740	0.727860	0.024844	O	0.222075	0.222782	0.286043
Sm	0.052740	0.227860	0.163449	O	0.472022	0.056147	0.286008
Sm	0.302740	0.061190	0.163449	O	0.721907	0.222701	0.286070
Sm	0.552740	0.227860	0.163449	O	0.972010	0.056015	0.286086
Sm	0.802740	0.061190	0.163449	O	0.222138	0.556243	0.286010
Sm	0.052740	0.561190	0.163449	O	0.472394	0.389356	0.286019
Sm	0.302740	0.394520	0.163449	O	0.722158	0.555867	0.286051
Sm	0.552740	0.561190	0.163449	O	0.972242	0.389235	0.286055
Sm	0.802740	0.394520	0.163449	O	0.221962	0.889617	0.286041
Sm	0.052740	0.894520	0.163449	O	0.472164	0.722775	0.285988
Sm	0.302740	0.727860	0.163449	O	0.722284	0.889149	0.286052
Sm	0.552740	0.894520	0.163449	O	0.971908	0.722884	0.286086
Sm	0.802740	0.727860	0.163449	O	0.152086	0.029370	0.319412
Sm	0.135137	0.063019	0.248893	O	0.402385	0.196022	0.319375
Sm	0.385157	0.229670	0.248869	O	0.652192	0.028878	0.319341
Sm	0.635140	0.062997	0.248864	O	0.902195	0.195927	0.319377
Sm	0.885162	0.229645	0.248888	O	0.152538	0.362755	0.319419
Sm	0.135162	0.396331	0.248901	O	0.402394	0.529113	0.319324
Sm	0.385185	0.563025	0.248863	O	0.652578	0.362529	0.319575
Sm	0.635205	0.396248	0.248922	O	0.902455	0.529039	0.319401
Sm	0.885179	0.562948	0.248905	O	0.152171	0.695869	0.319405
Sm	0.135132	0.729690	0.248898	O	0.402239	0.862461	0.319367
Sm	0.385144	0.896348	0.248885	O	0.652453	0.695821	0.319359
Sm	0.635161	0.729584	0.248866	O	0.902369	0.862731	0.319523
Sm	0.885184	0.896265	0.248914	O	0.222096	0.223305	0.353040
Sm	0.134796	0.063912	0.390448	O	0.471982	0.056595	0.352998
Sm	0.384670	0.230729	0.390274	O	0.722092	0.222748	0.353065
Sm	0.635125	0.063604	0.390303	O	0.971696	0.056669	0.353099
Sm	0.885215	0.230202	0.390388	O	0.222179	0.556662	0.353002
Sm	0.135328	0.397328	0.390536	O	0.472166	0.389940	0.353010

Sm	0.384672	0.564083	0.390220	O	0.722191	0.556628	0.353049
Sm	0.635085	0.397196	0.390506	O	0.972213	0.389765	0.353057
Sm	0.885267	0.564252	0.390336	O	0.221777	0.890022	0.353034
Sm	0.134827	0.730888	0.390413	O	0.472105	0.723165	0.352973
Sm	0.384892	0.897421	0.390352	O	0.722129	0.889887	0.353052
Sm	0.635134	0.730803	0.390279	O	0.971789	0.723083	0.353086
Sm	0.884584	0.897231	0.390658	O	0.052278	0.230197	0.407678
Sm	0.052712	0.230012	0.475825	O	0.301825	0.063527	0.407572
Sm	0.301880	0.063045	0.475721	O	0.552108	0.230151	0.407413
Sm	0.551677	0.228675	0.475124	O	0.801870	0.063535	0.407900
Sm	0.801473	0.064043	0.476287	O	0.052388	0.563509	0.407631
Sm	0.052792	0.563350	0.475782	O	0.302490	0.396927	0.407694
Sm	0.303307	0.396739	0.476112	O	0.552104	0.563273	0.407384
Sm	0.551588	0.564426	0.475107	O	0.802051	0.396713	0.407451
Sm	0.803423	0.396613	0.475197	O	0.051464	0.896869	0.407740
Sm	0.051781	0.896657	0.475823	O	0.301876	0.730336	0.407524
Sm	0.301899	0.730446	0.475670	O	0.551894	0.896686	0.407542
Sm	0.551730	0.896532	0.475753	O	0.801886	0.729911	0.407863
Sm	0.801539	0.728999	0.476261	O	0.135360	0.062981	0.458042
O	0.135710	0.061910	0.008726	O	0.385286	0.228991	0.457881
O	0.385710	0.228580	0.008726	O	0.634890	0.062697	0.457876
O	0.635710	0.061910	0.008726	O	0.886321	0.229837	0.457951
O	0.885710	0.228580	0.008726	O	0.135965	0.396577	0.458139
O	0.135710	0.395250	0.008726	O	0.385284	0.564343	0.457820
O	0.385710	0.561910	0.008726	O	0.635381	0.396502	0.458634
O	0.635710	0.395250	0.008726	O	0.886432	0.563088	0.457890
O	0.885710	0.561910	0.008726	O	0.135427	0.730280	0.457999
O	0.135710	0.728580	0.008726	O	0.385168	0.896681	0.457960
O	0.385710	0.895250	0.008726	O	0.634938	0.730147	0.457869
O	0.635710	0.728580	0.008726	O	0.884760	0.896446	0.458387
O	0.885710	0.895250	0.008726	O	0.218629	0.229409	0.512276
O	0.215390	0.235890	0.060814	O	0.468420	0.062685	0.512116
O	0.465390	0.069230	0.060814	O	0.721509	0.225060	0.512194
O	0.715390	0.235890	0.060814	O	0.969085	0.063076	0.512341
O	0.965390	0.069230	0.060814	O	0.218754	0.564035	0.512219
O	0.215390	0.569230	0.060814	O	0.461985	0.396894	0.512140
O	0.465390	0.402560	0.060814	O	0.721910	0.567899	0.512152
O	0.715390	0.569230	0.060814	O	0.969452	0.396619	0.512130
O	0.965390	0.402560	0.060814	O	0.218439	0.896712	0.512415
O	0.215390	0.902560	0.060814	O	0.468357	0.730428	0.512093
O	0.465390	0.735890	0.060814	O	0.718105	0.896448	0.512187
O	0.715390	0.902560	0.060814	O	0.969269	0.729833	0.512290
O	0.965390	0.735890	0.060814	C	0.237860	0.190940	0.094147
O	0.035180	0.262960	0.094147	C	0.487860	0.024280	0.094147

O	0.285180	0.096300	0.094147	C	0.737860	0.190940	0.094147
O	0.535180	0.262960	0.094147	C	0.987860	0.024280	0.094147
O	0.785180	0.096300	0.094147	C	0.237860	0.524280	0.094147
O	0.035180	0.596300	0.094147	C	0.487860	0.357610	0.094147
O	0.285180	0.429630	0.094147	C	0.737860	0.524280	0.094147
O	0.535180	0.596300	0.094147	C	0.987860	0.357610	0.094147
O	0.785180	0.429630	0.094147	C	0.237860	0.857610	0.094147
O	0.035180	0.929630	0.094147	C	0.487860	0.690940	0.094147
O	0.285180	0.762960	0.094147	C	0.737860	0.857610	0.094147
O	0.535180	0.929630	0.094147	C	0.987860	0.690940	0.094147
O	0.785180	0.762960	0.094147	C	0.199640	0.268110	0.319510
O	0.215390	0.235890	0.127479	C	0.449565	0.101379	0.319483
O	0.465390	0.069230	0.127479	C	0.699612	0.267805	0.319586
O	0.715390	0.235890	0.127479	C	0.949403	0.101310	0.319529
O	0.965390	0.069230	0.127479	C	0.199584	0.601404	0.319485
O	0.215390	0.569230	0.127479	C	0.449771	0.434602	0.319476
O	0.465390	0.402560	0.127479	C	0.699697	0.601218	0.319505
O	0.715390	0.569230	0.127479	C	0.949733	0.434467	0.319533
O	0.965390	0.402560	0.127479	C	0.199361	0.934821	0.319509
O	0.215390	0.902560	0.127479	C	0.449598	0.767952	0.319462
O	0.465390	0.735890	0.127479	C	0.699637	0.934450	0.319476
O	0.715390	0.902560	0.127479	C	0.949429	0.768071	0.319572
O	0.965390	0.735890	0.127479	H	0.717345	0.896733	0.540434
O	0.135710	0.061910	0.179568	H	0.220726	0.230886	0.540506
O	0.385710	0.228580	0.179568	H	0.218780	0.896724	0.540659
O	0.635710	0.061910	0.179568	H	0.965055	0.396800	0.540330
O	0.885710	0.228580	0.179568	H	0.470593	0.726185	0.540293
O	0.135710	0.395250	0.179568	H	0.470800	0.066978	0.540313
O	0.385710	0.561910	0.179568	H	0.221253	0.562816	0.540446
O	0.635710	0.395250	0.179568	H	0.967369	0.064412	0.540578
O	0.885710	0.561910	0.179568	H	0.967441	0.728212	0.540523
O	0.135710	0.728580	0.179568	Cu	0.688097	0.499109	0.555932
O	0.385710	0.895250	0.179568	Cu	0.533996	0.397115	0.555079
O	0.635710	0.728580	0.179568	Cu	0.687839	0.294584	0.555860
O	0.885710	0.895250	0.179568	Cu	0.635179	0.396890	0.611620
O	0.052144	0.229241	0.232122				

Table S2. The original data of the catalyst and intermediate.

species	single point energy / eV	thermal correction to Gibbs free energy (zero-point energy is included) / kJ· mol^{-1}				
		150 ℃	200 ℃	250 ℃	300 ℃	350 ℃
surface	-1901.535	512.524710	167.974014	-203.087947	-598.680193	-1017.081997
IM1	-1917.349	512.664360	164.219730	-210.939371	-610.817780	-1033.682506
IM2	-1916.940	529.381460	183.173978	-189.691465	-587.226517	-1007.703589
IM3	-1917.309	539.745399	194.284802	-177.802561	-574.537936	-994.200822
IM4	-1894.148	514.384505	172.193541	-196.409127	-589.458415	-1005.245869
IM5	-1909.134	562.622906	217.451552	-154.357269	-550.827168	-970.239532
IM6	-1909.894	558.175987	212.270581	-160.321706	-557.614054	-977.879494
IM7	-1909.395	551.652312	205.859486	-166.611079	-563.776514	-983.913269
CO	-14.798	-5.026086	-8.002838	-11.070473	-14.221634	-17.450435
CO_2	-22.989	28.675544	28.269461	27.783264	27.217739	26.574145
H_2O	-14.223	37.577973	34.262554	30.806889	27.221849	23.516320
H_2	-6.760	-82.147498	-95.678947	-109.290101	-122.973348	-136.722431

Table S3. Gibbs energy changes in reactions at the inlet of the WGS reaction unit. (partial pressures of CO and H_2O are 2000 Pa and 10000 Pa, respectively)

elementary reaction	Gibbs energy changes in reactions / eV				
	150 °C	200 °C	250 °C	300 °C	350 °C
surface + CO → IM1	-0.819	-0.811	-0.805	-0.800	-0.796
IM1 → IM2	0.582	0.606	0.629	0.654	0.679
IM2 → IM3	-0.261	-0.253	-0.245	-0.237	-0.228
IM3 → IM4 + CO_2	-0.215	-0.235	-0.254	-0.271	-0.287
IM4 +H_2O → IM5	-0.569	-0.555	-0.543	-0.531	-0.520
IM5 → IM6	-0.806	-0.813	-0.821	-0.830	-0.839
IM6 → IM7	0.431	0.432	0.433	0.435	0.436
IM7 → IM8 + H_2	-0.583	-0.760	-0.937	-1.113	-1.288

Table S4. The partial pressures of CO, H_2O, CO_2 and H_2 at air outlet.

Temperature (°C)	partial pressures (Pa)			
	CO	H_2O	CO_2	H_2
150	1530	9530	470	470
200	1154	9154	846	846
250	496	8496	1504	1504
300	60	8060	1940	1940
350	20	8020	1980	1980

Table S5. Gibbs energy changes in reactions at the outlet of the WGS reaction unit.

elementary reaction	Gibbs energy changes in reactions / eV				
	150 °C	200 °C	250 °C	300 °C	350 °C
surface + CO → IM1	-0.809	-0.789	-0.742	-0.626	-0.548
IM1 → IM2	0.582	0.606	0.629	0.654	0.679
IM2 → IM3	-0.261	-0.253	-0.245	-0.237	-0.228
IM3 → IM4 + CO_2	0.198	0.227	0.255	0.280	0.309
IM4 +H_2O → IM5	-0.567	-0.552	-0.535	-0.520	-0.508
IM5 → IM6	-0.806	-0.813	-0.821	-0.830	-0.839
IM6 → IM7	0.431	0.432	0.433	0.435	0.436
IM7 → IM8 + H_2	-0.166	-0.294	-0.422	-0.549	-0.675

Table S6 Physical properties of the obtained samples.

Samples	Cu loading [a] (wt%)	S_{BET} [b] (m^2□g^{-1})	V_{total} [$cm^3\ g^{-1}$]	BJH pore size [nm]
$Sm_2O_2CO_3$	–	95	1.62	34.29
$5Cu/Sm_2O_2CO_3$	3.8	83	1.10	26.70
$10Cu/Sm_2O_2CO_3$	8	73	1.38	22.01
$20Cu/Sm_2O_2CO_3$	19.8	77	1.00	27.38
Al_2O_3	–	300	0.59	3.92
$5Cu/Al_2O_3$	7.3	267	0.66	4.95
CeO_2	–	66	0.30	9.24
$5Cu/CeO_2$	7.5	47	0.38	16.03

[a]Determined by ICP-AES; [b, c]BET surface area of samples

Table S7 The Cu dispersion over various $Cu/Sm_2O_2CO_3$ catalysts from H_2-TPR.

Samples	Peak area-N_2O	Peak area-Air	Dispersion (%)
$5Cu/Sm_2O_2CO_3$	894	1559	100
$10Cu/Sm_2O_2CO_3$	1386	3090	90
$20Cu/Sm_2O_2CO_3$	1522	5277	58

Table S8. The decomposition of H_2O molecule on the surface of catalyst through the redox mechanism. (surface + H_2O→ *OH_2; *OH_2 →*OH + *H; *OH + *H → *O + *H + *H)

decomposition reaction	Gibbs energy changes in reactions / eV				
	150 °C	200 °C	250 °C	300 °C	350 °C
*OH_2 →*OH + *H	-0.459	-0.431	-0.403	-0.374	-0.346
*OH + *H → *O + *H + *H	1.589	1.600	1.611	1.623	1.635

References

(1) Kresse, G.; Hafner, J. Ab. initio molecular dynamics for liquid metals. *Phys. Rev. B.* **1993**, *47*, 558–561.

(2) Kresse, G.; Furthmuller, J. Efficient iterative schemes for ab initio total-energy calculations using a plane-wave basis set. *Phys. Rev. B.* **1996**, *54*, 11169−11186.

(3) Togo, A.; Tanaka, I. First principles phonon calculations in materials science. *Scripta Mater.* **2015**, *108*, 1–5.
